# New Indole Glycosides from *Aesculus chinensis* var. *chekiangensis* and Their Neuroprotective Activities

**DOI:** 10.3390/molecules24224063

**Published:** 2019-11-09

**Authors:** Nan Zhang, Shijie Cao, Weixing Huang, Pan Li, Ning Kang, Liqin Ding, Feng Qiu

**Affiliations:** 1 School of Chinese Materia Medica, Tianjin University of Traditional Chinese Medicine, Tianjin 301617, China; 13373750625@163.com (N.Z.); satellitehuang@163.com (W.H.); a_pan_18@126.com (P.L.); kangndd@163.com (N.K.); 2 Tianjin State Key Laboratory of Modern Chinese Medicine, Tianjin University of Traditional Chinese Medicine, Tianjin 301617, China; caoshijie@tjutcm.edu.cn

**Keywords:** *Aesculus chinensis* Bge. var. *chekiangensis* (Hu et Fang) Fang, *N*-glucosylated indoles, neuroprotective activity

## Abstract

The dried seeds of *Aesculus chinensis* Bge. var. *chekiangensis* (Hu et Fang) Fang, called “Suo Luo Zi”, have been used in traditional Chinese medicine. Nevertheless, most studies have been focused on components of less polarity fractions. In this research, twelve indoles, including six new indole glycosides (**1**–**6**) as well as six known analogs were isolated from the polar portion which has been seldom studied. This is the first description of N-glucosylated indoles obtained from the genus of *Aesculus*. Structures of the new compounds (**1**–**6**) were elucidated based on comprehensive interpretation of HRESIMS, 1D and 2D NMR. Additionally, the neuroprotective activities of the N-glucosylated indoles were evaluated for the first time indicating that compounds **1**–**5** and **9**–**10** exhibited moderate neuroprotective activities. Further cytotoxicity tests of isolates **1**–**10** on three human tumor cell lines suggested that none of these compounds were cytotoxic (IC_50_ > 50 μM).

## 1. Introduction

**Figure 1:**
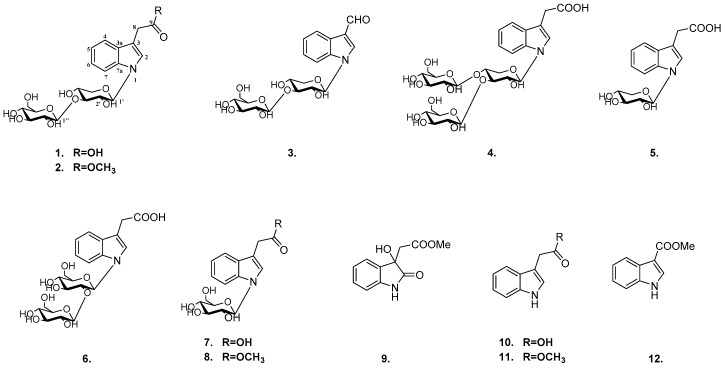
The structures of compounds **1**–**12**.

*Aesculus chinensis* Bge. var. *chekiangensis* (Hu et Fang) Fang belonging to the *Hippocastanaceae* family is a species endemic to China. Its dried seeds together with *Aesculus chinensis* Bge. and *Aesculus wilsonii* Rehd have been used in traditional Chinese medicine for treating chest and abdomen pain, dysentery and ague [1,2]. Recently, many studies have demonstrated that *Aesculus chinensis* have beneficial effects involving their antitumor, cardio-protective, anti-inflammatory, and neuroprotective activities [3,4]. Previous investigation of its chemical constituents resulted in various types of compounds, such as triterpenoids [5,6,7], flavonoids [8,9], coumarins [10] and steroids. So far, most studies have been focused on components of less polarity fractions and little has been known about polar fractions consists. As an extension study on biologically active compounds from polar portion, six new indole glycosides (**1**–**6**) and six known analogs (**7**–**12**) were obtained (Figure 1) and their neuroprotective activities and cytotoxic activities were also evaluated.

## 2. Results

Compound **1** was isolated as a yellow, amorphous powder (MeOH). The UV spectrum showed absorption at 290 nm, and its pseudomolecular ion [M+H]^+^ at *m/z* 470.1655, indicating the presence of odd number of nitrogen atom in compound **1**. The HRMS analysis (*m/z* 470.1655 [M + H]^+^) and the NMR data (Table 1 and Table 2) indicated the molecular formula C_21_H_27_NO_11_.

The ^13^C-NMR spectrum exhibited resonances of 21 carbon signals composed of nine unsaturated carbons (δ_C_ 176.0, 138.2, 129.8, 126.4, 122.9, 120.9, 119.8, 111.7, 109.8), eleven sugar carbons (δ_C_ 105.0, 86.1, 81.1, 79.0, 77.7, 77.4, 75.8, 70.9, 70.8, 69.4, 62.0) and one additional aliphatic carbons (*δ*_C_ 31.9). The ^1^H-NMR spectrum displayed a spin coupling system of four aromatic protons [δ_H_ 7.53 (1H, d, *J* = 7.8 Hz), 7.48 (1H, d, *J* = 8.3 Hz), 7.16 (1H, ddd, *J* = 8.2, 7.0, 1.2 Hz), 7.08 (1H, ddd, *J* = 7.5, 7.0, 0.9 Hz)] indicating an ortho-substituted aromatic ring; an additional aromatic proton signal at δ_H_ 7.41 (1H, s), indicative of a 3-substituted indole moiety, an isolated methylene protons at *δ*_H_ 3.72 (2H, s) and two anomeric protons at *δ*_H_ 5.45 (1H, d, *J* = 9.0 Hz) and 4.35 (1H, d, *J* = 7.8 Hz).

All of the protons and carbons were unambiguously assigned by HSQC experiment.

The HMBC spectrum correlations between H-4 (*δ*_H_ 7.53, 1H, d, *J* = 7.8 Hz) and C-3 (*δ*_C_ 109.8), C-6 (δ_C_ 122.9), C-7a (δ_C_ 138.2); between H-5 (δ_H_ 7.08, 1H, ddd, *J* = 7.5, 7.0, 0.9 Hz) and C-7 (δ_C_ 111.7), C-3a (δ_C_ 129.8); between H-6 (δ_H_ 7.16, 1H, ddd, *J* = 8.2, 7.0, 1.2 Hz) and C-4 (δ_C_ 119.8), C-7a (δ_C_ 138.2) and between H-7 (δ_H_ 7.48, 1H, d, *J* = 8.3 Hz) and C-5 (δ_C_ 120.9), C-3a (δ_C_ 129.8) further confirm the existence of the ortho-substituted aromatic ring in compound **1**. The substituent at C(3) was deduced to be a CH_2_COOH group, supported by the HMBC correlations between H-2 (δ_H_ 7.41, 1H, s) and C-3a (*δ*_C_ 129.8), C-7a (δ_C_ 138.2), C-8 (δ_C_ 31.9) and between H-8 (δ_H_ 3.72) and C-2 (δ_C_ 126.4), C-3 (δ_C_ 109.8), C-3a (δ_C_ 129.8), C9 (δ_C_ 176.0). Thus, the aglycone of compound **1** was established as indole-3-acetic acid.

**Figure 2:**
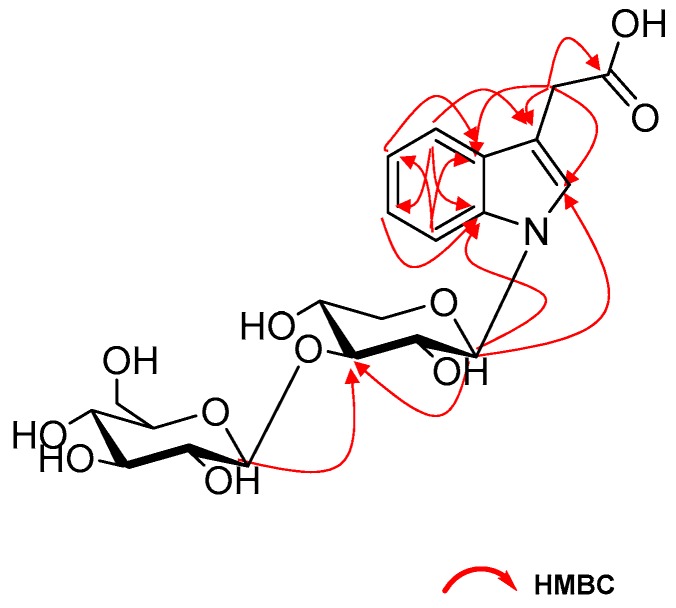
The selected HMBC (H→C) correlations of compound **1**.

The two anomeric protons at *δ* 5.45 (1H, d, *J* = 9.0 Hz), 4.35 (1H, d, *J* = 7.8 Hz) correlated with carbons at *δ* 86.1 and 105.0 in HSQC spectrum, respectively, indicated disaccharide residues. Acid hydrolysis of **1** with 2M HCl produced d-glucose and d-xylose, which was identified with HPLC analysis by comparing with authentic sugar samples after derivatization [11]. The *β*-configuration of the glycosidic linkages were deduced from the large coupling constants. In addition, the HMBC correlations of H-1′ (δ 5.45) with C-2, C-7a and H-1″ (δ 4.35) with C-3′ revealed the sequence glc-(1→3)-xyl to be linked at nitrogen of aglycone portion (Figure 2). Based on the above analyses, the structure of **1** was identified as *N*-[*β*-d-glucopyranosyl(1→3)]*-β*-d-xylopyranosyl-indole-3-acetic acid.

Compound **2** was obtained as a yellow amorphous powder with the elemental formula C_22_H_29_NO_11_ (HR-ESI-MS *m/z*: 484.1811 [M + H]^+^; calculated for C_22_H_30_NO_11_, 484.1819). Acid hydrolysis of **2** yielded the same sugar units as **1**. Its NMR spectra were closely similar to those of **1** with the only difference of an extra methoxy signals (*δ*_H_ 3.71 and *δ*_C_ 52.5). The HMBC cross-peaks of H-10 (δ_H_ 3.71) with C-9 (δ_C_ 174.4) implied the -COOH in **1** was replaced by -COOCH_3_ in **2**, which was further confirmed by their formulas. Thus, compound **2** was assigned as *N*-[*β*-d-glucopyranosyl(1→3)]*-β*-d- xylopyranosyl-indole-3-methyl acetate.

Compound **3**, a yellow, amorphous powder, was assigned the molecular formula C_20_H_25_NO_10_ (HRESIMS *m/z* 440.1549 [M + H]^+^; calculated for C_20_H_26_NO_10_, 440.1557). The sugar chain of **3** was the same as that of **2** by comparing their ^1^H and ^13^C-NMR data (Table 1 and Table 2) and analysis of the hydrolysis result. The similar NMR spectra of **3** to those of **1** and **2** indicated that compound **3** is a structural analogue of these compounds. The ^1^H-NMR spectra showed proton resonances corresponding to a 3-substituted indole group [δ_H_ 8.18 (1H, d, *J* = 7.7 Hz), 7.62 (1H, d, *J* = 8.1 Hz), 7.31 (1H, ddd, *J* = 8.1, 7.3, 1.1 Hz), 7.26 (1H, ddd, *J* = 7.7, 7.3, 0.9 Hz), 8.37 (1H, s)]. The substituent at C-3 was deduced as a formyl moiety, established by HSQC correlations between H-8 (*δ*_H_ 3.71) and C-8 (δ_C_ 187.7) together with the HMBC correlations between H-8 (δ_H_ 3.71) and C-3 (δ_C_ 119.7) as well as C-3a (δ_C_ 126.4). Hence, **3** was defined as *N*-[*β*-d-glucopyranosyl(1→3)]*-β*-d-xylopyranosyl-indole-3-carbaldehyde.

Compound **4** was assigned the molecular formula of C_27_H_37_NO_16_ from HRESIMS (*m*/*z* 632.2150 [M + H]^+^*,* calculated for 632.2191) and its NMR data. The MS and NMR spectra were closely parallel to those of **1**, revealing the same aglycone and sugar residues with the difference of an additional hexose unit (162 Da) in compound **4**. This conclusion was further ensured by hydrolysis, conversion to chiral diastereomers and HPLC analysis. The HMBC correlations from H-1′ (δ 5.46) to C-2 (δ 126.4), C-7a (δ138.2), from H-1″ (δ 4.37) to C-3′ (δ 80.5) and from H-1‴ (δ 4.43) to C-4′ (δ 77.6) confirmed the linkage of the trisaccharide moiety in **4**. Consequently, it was assigned as *N*-[*β*-d-glucopyranosyl(1→3)-[*β*-d-glucopyranosyl (1→4)]*-β*-d-xylopyranosyl-indole-3-acetic acid.

For compound **5**, isolated as a yellowish powder, established the molecular formula was C_15_H_17_NO_6_ by HRESIMS (*m*/*z* 308.1136 [M + H]^+^; calculated for C_15_H_18_NO_6_, 308.1134). Acid hydrolysis of **5** yielded d-xylose, which was identified using the same method as **1**–**4**. Analysis of the ^1^H- and ^13^C-NMR spectroscopic data (Table 1 and Table 2) of **5** displayed a close structural resemblance to **1**, except for the absence of a d-glucose. This deduction was supported by the key HMBC correlations from H-1′ (δ 5.31) to C-2 (*δ* 125.0), C-7a (δ 138.3) and from H-8 (δ 3.66) to C-2 (δ 125.0), C-3 (δ112.2), C3a (δ130.2), C9 (δ180.1). Accordingly, **5** was unambiguously established as *N-β*-d-xylopyranosyl-indole-3-acetic acid.

Compound **6** had a molecular formula of C_22_H_29_NO_12_, which was established from the [M+H]^+^ ion at *m*/*z* 500.1762 (calculated for 500.1768) in the positive HR-ESI-MS, 30 mass units more than that of **1**. Comparison of the NMR data of **6** with those of **1** showed that both isolates are closely related, but only differed at the disaccharide group. Acid hydrolysis suggested that only d-glucose existed in **6**. As observed in the HMBC spectrum, the long-range correlations of H-1′ (δ 5.56) with C-2 (δ 126.4), C-7a (δ138.4) and H-1″ (δ 4.38) with C-2′ (δ 81.2) provided definitive evidences that the linkage glc-(1→2)-glc was bound to nitrogen of aglycone portion. Therefore, compound **6** was defined as *N*-[*β*-d-glucopyranosyl (1→2)]*-β*-d-xylopyranosyl-indole-3-acetic acid.

The known indoles were identified as *N*-*β*-d-glucopyranosyl-indole-3-acetic acid (**7**) [12], *N*-*β*-d-glucopyranosyl-indole-3-methyl acetate (**8**) [13], methyl dioxindole-3-acetate (**9**) [14], indole-3-acetic acid (**10**) [15], indole-3-methyl acetate (**11**) [15], indole-3-carboxylic acid methyl ester (**12**) [16] by NMR analysis and comparison with literature data.

**Figure 3:**
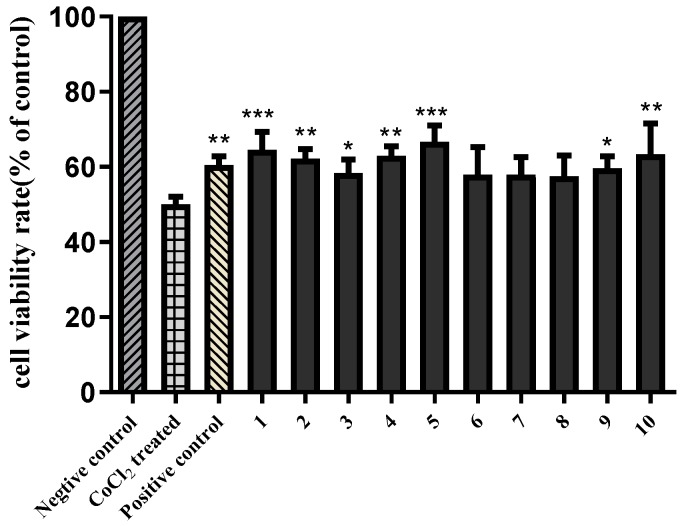
Neuroprotective activities of compounds **1**–**10** (10 μM) against C_O_Cl_2_-induced cell death in PC12 cells. The data (cell viability, measured by MTT assay) are expressed as means ± SD. Three independent experiments were performed. Trolox was used as the positive control at 10 μM. Compared with CoCl_2_ treated group, * *p* < 0.05, ** *p* < 0.01, *** *p* < 0.001.

It has been reported that *Aesculus chinensis* showed neuroprotective activities [3]. In the present study, the isolated compounds **1**−**10** were also evaluated for their neuroprotective effects against CoCl_2_-induced PC12 cell damage. As shown in Figure S43, all substances showed no obvious cytotoxic effects on PC12 cells at a dose of 10 μM. Then, 10 μM samples were bioassayed for neuroprotective activities against CoCl_2_-induced toxicity in PC12 cells with Trolox (10 μM) as the positive control. According to Figure 3, compared with the Trolox, compounds **1**–**5** and **9–10** show similar effect on increasing the cell viabilities in CoCl_2_-treated PC12 cells, indicating that compounds **1**–**5** and **9**–**10** exhibited statistically significant neuroprotective activities.

The cytotoxic activities against three human cancer cell lines (Hep G2, HCT-116, and MGC-803) of compounds **1**–**10** were assayed using the MTT method, with 5-fluorouracil (5-FU) as the positive control. None of these compounds displayed cytotoxic activity (IC_50_ > 50 μM) (Table S1).

## 3. Materials and Methods

### 3.1. General Experimental Procedures

Optical rotations were recorded on a Rudolph (Hackettstown, NJ, USA) Autopol V automatic polarimeter. The UV spectra were acquired on a UNICO 2102PCS spectrophotometer (Dayton, NJ, USA). The IR spectra were obtained in a KBr-disc (cm^−1^) on a Brucker Tensor II spectrometer (Billerica, MA, USA). NMR spectra were carried out on a Bruker (Billerica, MA, USA) AM-600 spectrometer at 25 °C referencing to the residuals of CD_3_OD. High-Resolution-ESI-MS (HR-ESI-MS) was performed on a Waters (Milford, MA, USA) Xevo G2-S UPLC-Q/TOF equipped with an ACQUITY UPLC BEH C18 (2.1 × 50 mm, Waters 1.7 μm, Milford, MA, USA). Analytical HPLC were performed on a Waters e2695 system equipped with a 2998 PDA detector (Waters, Milford, MA, USA) using a YMC-Pack-ODS-A column (250 × 4.6 mm, 5 μm, YMC, Tokyo, Japan). Semi-preparative HPLC was performed using a Shimadzu LC-6AD Series instrument equipped with a YMC Packed C_18_ column (250 × 10.0 mm, 5 μm, YMC, Tokyo, Japan) and detected with a DAD detector (Shimadzu, Tokyo, Japan) set at 205 and 230 nm. Column chromatography (CC) was done with Sephadex LH-20 (GE Healthcare Co. Ltd., Marlborough, MA, USA), ODS RP-C_18_ (40–75 μm Merck, Darmstadt, Germany), macroporous resin D101 (Chemical Plant of Nankai University, Tianjin, China), and silica gel (200–400 mesh, Qingdao Haiyang Chemical, Qingdao, China). All reagents used were of analytical grade (Concord Technology Co. Ltd., Tianjin, China).

### 3.2. Plant Material

Seeds of *Aesculus chinensis* Bge. var. *chekiangensis* (Hu et Fang) Fang were purchased from An guo (Hebei Province, China) in August 2015, and authenticated by professor Lijuan Zhang (Tianjin University of Traditional Chinese Medicine, Tianjin, China). A voucher specimen was deposited at the School of Chinese Materia Medica, Tianjin University of Traditional Chinese Medicine.

### 3.3. Extraction and Isolation

The dried seeds of *A. chinensis* Bge. (8.8 kg) were cut into small pieces and were extracted with 70% ethanol three times (3 h) under reflux. After removal of the solvent under reduced pressure, a dark residue (2100 g) was afforded. The residue was suspended in H_2_O and subjected to D101 resin and then sequentially eluted with H_2_O, a gradient of EtOH in water to yield the corresponding fractions. The 20% EtOH−H_2_O part was further fractionated with a silica gel column, eluting with a gradient of 0–100% CH_2_Cl_2_/CH_3_OH to yield 4 fractions (A–D).

Fraction A (8.0 g) was applied to an RP C_18_ CC (MeOH−H_2_O, from 0:100 to 50:50) to give four subfractions (A1−A4). Subfraction A2 was purified by an RP-HPLC (MeCN−H_2_O, 8:92, 3.0 mL/min) to obtain compounds **9** (6.7 mg, *t*_R_ 11.2 min) and **12** (5.4 mg, *t*_R_ 14.7 min). Further purification of subfraction A3 using preparative RP-HPLC (MeCN−H_2_O, 8:92, 3.0 mL/min) yielded compounds **10** (4.8 mg, *t*_R_ 16.5 min) and **11** (2.3 mg, *t*_R_ 17.8 min).

Fraction C (22.0 g) was applied to an ODS MPLC column eluting with gradient MeOH−H_2_O from 10:90 to 100: 0 to afford five major subfractions (C1–C5). Compound **4** (9.2 mg, *t*_R_ 31.5 min) was purified by preparative HPLC with 10% MeCN/H_2_O from subfraction C1. Compounds **1** (7.1 mg, *t*_R_ 8.9 min), **2** (13.5 mg, *t*_R_ 9.7 min), **3** (11.0 mg, *t*_R_ 13.4 min) and **6** (9.6 mg, *t*_R_ 16.5 min) were obtained from Fr. C2 using Sephadex LH-20 column and further purified by RP-HPLC (MeCN−H_2_O 15: 85, *v*/*v*, 3.0 mL/min). Subfraction C3 was purified by preparative HPLC to afford compounds **7** (7.0 mg, *t*_R_ 16.5 min) and **8** (9.1 mg, *t*_R_ 18.2 min) using 15% MeCN/H_2_O. Subfraction C4 was chromatographed on a Sephadex LH-20 column and then purified through preparative HPLC with 15% MeCN/H_2_O to yield compound **5** (9.8 mg, *t*_R_ 20.3 min).

#### 3.3.1. *N*-[β-d-glucopyranosyl(1→3)]-β-d-xylopyranosyl-indole-3-acetic Acid (**1**)

Yellow amorphous powder; ${\left[\alpha \right]}_{D}^{25}$ – 3.2 (*c* 0.1, MeOH); ^1^H-NMR and ^13^C-NMR: Table 1 and Table 2; HR-ESI-MS: *m/z* 470.1655 [M + H]^+^ (calculated for C_21_H_28_NO_11_, 470.1662).

#### 3.3.2. *N*-[β-d-glucopyranosyl(1→3)]-β-d-xylopyranosyl-indole-3-methyl Acetate (**2**)

Yellow amorphous powder; ${\left[\alpha \right]}_{D}^{25}$ – 2.0 (*c* 0.10, MeOH); ^1^H-NMR and ^13^C-NMR: Table 1 and Table 2; HR-ESI-MS: *m/z* 484.1811 [M + H]^+^ (calculated for C_22_H_30_NO_11_, 484.1819).

#### 3.3.3. *N*-[β-d-glucopyranosyl(1→3)]-β-d-xylopyranosyl-indole-3-carbaldehyde (**3**)

Yellow amorphous powder; ${\left[\alpha \right]}_{D}^{25}$ – 2.2 (*c* 0.11, MeOH); ^1^H-NMR and ^13^C-NMR: Table 1 and Table 2; HR-ESI-MS: *m/z* 440.1549 [M + H]^+^ (calculated for C_20_H_26_NO_10_, 440.1557).

#### 3.3.4. *N*-[β-d-glucopyranosyl(1→3)-[β-d-glucopyranosyl(1-4)]-β-d-xylopyranosyl-indole-3-acetic Acid (**4**)

Yellow amorphous powder; ${\left[\alpha \right]}_{D}^{25}$ – 6.3 (*c* 0.10, MeOH); ^1^H-NMR and ^13^C-NMR: Table 1 and Table 2; HR-ESI-MS: *m/z* 632.2150 [M + H]^+^ (calculated for C_27_H_38_NO_16_, 632.2191).

#### 3.3.5. *N*-β-d-xylopyranosyl-indole-3-acetic Acid (**5**)

Yellowish powder; ${\left[\alpha \right]}_{D}^{25}$ – 2.0 (*c* 0.12, MeOH); ^1^H-NMR and ^13^C-NMR: Table 1 and Table 2; HR-ESI-MS: *m/z* 308.1136 [M + H]^+^ (calcd. for C_15_H_18_NO_6_, 308.1134).

#### 3.3.6. *N*-[β-d-glucopyranosyl(1→2)]-β-d-xylopyranosyl-indole-3-acetic Acid (***6***)

Yellowish powder; ${\left[\alpha \right]}_{D}^{25}$ – 4.8 (*c* 0.09, MeOH); ^1^H-NMR and ^13^C-NMR: Table 1 and Table 2; HR-ESI-MS: *m/z* 500.1762 [M + H]^+^ (calculated for C_22_H_30_NO_12_, 500.1768).

#### 3.3.7. Hydrolysis and Determination of Absolute Configuration of Sugars

A solution of **1**–**6** (1.0 mg, respectively) dissolved in 2 M HCl (4.0 mL) was heated at 90 °C for 2 h. The reaction mixture was extracted two times with EtOAc (4 mL), and the aqueous layer was evaporated to dryness under N_2_ atmosphere. Then l-cysteine methyl ester (1.0 mg) was added to the residues dissolved in pyridine (1.0 mL) and heated at 60 °C. One hour later, *o*-tolyisothiocyanate (1.0 mL) was added and heated for another hour. Then each reaction mixture was analyzed by the Waters e2695 HPLC system (YMC- Pack-ODS-A column, 1.0 mL/min, 250 nm) eluting with A (0.1% formic acid): B (acetonitrile) = 80: 20 (*v*/*v*). By comparison of the retention times with the standards, the absolute configuration of sugars in **1**–**6** was established [11,17].

### 3.4. Neuroprotective Effect Assay

The neuroprotective effects of compounds **1**–**10** was evaluated on CoCl_2_ damaged PC12 cells model [18,19]. Rat pheochromocytoma cell line (PC12) were cultured in RPMI-1640 medium with 10% (*v*/*v*) inactivated fetal bovine serum and 100 U/mL penicillin/streptomycin. The cells were grown and treated at 37 °C in 5% CO_2_ and 95% humidified air incubator. Cells were placed into a 96-well plate at a density of 2 × 10^4^ cells/well and kept there for 24 h for the adherence of the cells. Cells were treated with the compounds at concentrations of 10 μM for 2 h. After incubation, 1 mM CoCl_2_ was added and incubated for 24 h. After a 24 h treatment, the supernatant was changed with MTT solution (5 mg/mL). After incubation at 37 °C for 4 h, cells were finally lysed with 150 μL of DMSO. The absorbance was measured at 490 nm with a microplate reader. The cell viability was indicated as a percentage of the live control cells. The results were expressed as means ± SD of the indicated numbers from three independent experiments. Statistical analysis was performed by one-way analysis of variance (ANOVA) and Student’s Dunnett test using the SPSS statistical software (version 19 for Windows, IBM Corp., Armonk, NY, USA). P values below 0.05 were considered statistically significant.

### 3.5. Cytotoxicity Assay

The human cancer cell lines, HepG2, HCT-116, and MGC-803 were purchased from ATCC. The in vitro cytotoxicity of compounds **1**–**10** was tested by MTT assay [20,21] with 5-fluorouracil as the positive control. The tested cell lines were cultured in 96-well plates at a density of 1 × 10^4^ cells/well and incubated for 24 h. Subsequently, cells were treated with compounds **1**–**10** at a dosage of 3.125–50 μM, respectively. After 24 h, the supernatant was changed with MTT solution (5 mg/mL) and incubated for another 4 h. Then cells were finally lysed with 150 μL of DMSO and the absorbance was measured at 490 nm with a microplate reader. The cell viability was indicated as a percentage of the live control cells.

## 4. Conclusions

In summary, this is the first study of the water-soluble portion of *Aesculus chinensis* Bge. var. *chekiangensis* (Hu et Fang) Fang, six new indole glycosides (**1**–**6**) along with six known analogs were isolated and characterized. What is more, this is the first report of *N*-glucosylated indoles from *Aesculus* genus, which largely enriched its chemical diversity. In addition, the neuroprotective activities of the *N*-glucosylated indoles were evaluated for the first time and compounds **1**–**5**, **9**–**10** exhibited statistically significant neuroprotective activities.

## Figures and Tables

**Table 1:** ^1^H-NMR spectroscopic data (*δ*) for compounds **1**–**6**^a^ (δ in ppm, *J* in Hz).

NO.	1	2	3	4	5	6
2	7.41 (1H, s)	7.44 (1H, s)	8.37 (1H, s)	7.41 (1H, s)	7.25 (1H, s)	7.47 (1H, s)
4	7.53 (1H, d, *J* = 7.8 Hz)	7.54 (1H, d, *J* = 7.8 Hz)	8.18 (1H, d, *J* = 7.7 Hz)	7.53 (1H, d, *J* = 7.8 Hz)	7.54 (1H, d, *J* = 7.9 Hz)	7.54 (1H, d, *J* = 7.9 Hz)
5	7.08 (1H, ddd, *J* = 7.5, 7.0, 0.9 Hz)	7.11 (1H, ddd, *J* = 7.5, 7.0, 0.9 Hz)	7.26 (1H, ddd, *J* = 7.7, 7.3, 0.9 Hz)	7.08 (1H, ddd, *J* = 7.5, 7.0, 1.2 Hz)	7.06 (1H, ddd, *J* = 7.9, 7.0, 0.9 Hz)	7.08 (1H, ddd, *J* = 7.5, 7.0, 1.2 Hz)
6	7.16 (1H, ddd, *J* = 8.2, 7.0, 1.2 Hz)	7.19 (1H, ddd, *J* = 8.2, 7.0, 1.1 Hz)	7.31 (1H, ddd, *J* = 8.1, 7.3, 1.1 Hz)	7.16 (1H, ddd, *J* = 8.2, 7.0, 1.2 Hz)	7.15 (1H, ddd, *J* = 8.2, 7.0, 1.1 Hz)	7.17 (1H, ddd, *J* = 8.2, 7.0, 1.2 Hz)
7	7.48 (1H, d, *J* = 8.3 Hz)	7.51 (1H, d, *J* = 8.3 Hz)	7.62 (1H, d, *J* = 8.1 Hz)	7.48 (1H, d, *J* = 8.2 Hz)	7.48 (1H, d, *J* = 8.3 Hz)	7.51 (1H, d, *J* = 8.3 Hz)
8	3.72 (2H, s)	3.79 (2H, s)	9.91 (1H, s)	3.71 (2H, s)	3.66 (2H, s)	3.72 (2H, s)
10		3.71 (3H, s)				
	Xyl-*p*	Xyl-*p*	Xyl-*p*	Xyl-*p*	Xyl-*p*	Glc-*p*
1′	5.45 (1H, d, *J* = 9.0 Hz)	5.48 (1H, d, *J* = 9.0 Hz)	5.58 (1H, d, *J* = 9.0 Hz)	5.46 (1H, d, *J* = 9.0 Hz)	5.31 (1H, d, *J* = 9.0 Hz)	5.56 (1H, d, *J* = 9.0 Hz)
2′	3.75 (1H, m)	3.78 (1H, m)	3.80 (1H, m)	3.93 (1H, d, *J* = 8.8 Hz)	3.89 (1H, t, *J* = 9.0 Hz)	4.21 (1H, t, *J* = 9.0 Hz)
3′	4.19 (1H, t, *J* = 8.8 Hz)	4.21 (1H, t, *J* = 8.7 Hz)	4.26 (1H, t, *J* = 8.6 Hz)	4.26 (1H, t, *J* = 8.7 Hz)	3.54 (1H, t, *J* = 9.0 Hz)	3.82 (1H, t, *J* = 8.8 Hz)
4′	3.74 (1H, m)	3.76 (1H, m)	3.78 (1H, m)	3.96 (1H, m)	3.69 (1H, ddd, *J* = 10.6, 9.0, 5.5 Hz)	3.54 (1H, m)
5′	3.48 (1H, m) 3.97 (1H, m)	3.50 (1H, m) 3.99 (1H, dd, *J* = 11.4, 4.7 Hz)	3.56 (1H, t, *J* = 10.5 Hz) 4.06 (1H, dd, *J* = 11.3, 4.7 Hz)	3.56 (1H, d, *J* = 10.8 Hz) 4.15 (1H, dd, *J* = 11.6, 5.1 Hz)	3.48 (1H, t, *J* = 11.0 Hz) 3.97 (1H, dd, *J* = 11.3, 5.5 Hz)	3.56 (1H, m)
6′						3.70 (1H, m) 3.86 (1H, dd, *J* = 12.2, 2.0 Hz)
	Glc-*p*	Glc-*p*	Glc-*p*	Glc-*p*		Glc-*p*
1″	4.35 (1H, d, *J* = 7.8 Hz)	4.38 (1H, d, *J* = 7.8 Hz)	4.48 (1H, d, *J* = 7.8 Hz)	4.37 (1H, d, *J* = 7.8 Hz)		4.38 (1H, d, *J* = 7.8 Hz)
2″	2.95 (1H, dd, *J* = 9.3, 7.8 Hz)	2.98 (1H, dd, *J* = 9.3, 7.8 Hz)	2.89 (1H, m)	2.95 (1H, dd, *J* = 9.3, 7.8 Hz)		2.96 (1H, dd, *J* = 9.3, 7.8 Hz)
3″	3.17 (1H, t, *J* = 9.1 Hz)	3.20 (1H, t, *J* = 8.5 Hz)	3.16 (1H, d, *J* = 8.9 Hz)	3.17 (1H, d, *J* = 9.1 Hz)		3.20 (1H, t, *J* = 9.1 Hz)
4″	3.05 (1H, t, *J* = 9.4 Hz)	3.06 (1H, t, *J* = 9.4 Hz)	2.92 (1H, m)	3.04 (1H, t, *J* = 9.4 Hz)		3.05 (1H, t, *J* = 9.4 Hz)
5″	2.74 (1H, ddd, *J* = 9.8, 4.6, 2.5 Hz)	2.77 (1H, ddd, *J* = 9.7, 4.7, 2.6 Hz)	2.87 (1H, m)	2.73 (1H, ddd, *J* = 9.9, 4.6, 2.5 Hz)		2.77 (1H, ddd, *J* = 9.8, 4.5, 2.5 Hz)
6″	3.10 (1H, dd, *J* = 11.8, 2.5 Hz) 3.21 (1H, dd, *J* = 11.7, 4.6 Hz)	3.13 (1H, dd, *J* = 11.7, 2.6 Hz) 3.23 (1H, dd, *J* = 11.1, 4.1 Hz)	3.16 (1H, d, *J* = 8.9 Hz) 3.35 (1H, dd, *J* = 11.6, 2.5 Hz)	3.10 (1H, dd, *J* = 11.8, 2.5 Hz) 3.21 (1H, dd, *J* = 12.2, 4.8 Hz)		3.14 (1H, dd, *J* = 11.8, 2.5 Hz) 3.23 (1H, dd, *J* = 11.8, 4.6 Hz)
				Glc-*p*		
1‴				4.43 (1H, d, *J* = 7.8 Hz)		
2‴				3.26 (1H, dd, *J* = 9.2, 7.8 Hz)		
3‴				3.35 (1H, m)		
4‴				3.29 (1H, m)		
5‴				3.33 (1H, m)		
6‴				3.67 (1H, dd, *J* = 12.0, 5.9 Hz) 3.89 (1H, dd, *J* = 12.4, 2.5 Hz)		

^a^ NMR data (δ) were measured at 600 MHz in CD_3_OD for **1**–**6**.

**Table 2:** ^13^C-NMR spectroscopic data (*δ*) for compounds **1**–**6**^a^ (*δ* in ppm).

NO.	1	2	3	4	5	6
2	126.4	126.5	141.4	126.4	125.0	126.4
3	109.8	109.4	119.7	109.9	112.2	109.7
3a	129.8	129.6	126.4	129.8	130.2	129.7
4	119.8	119.8	122.5	119.9	120.0	119.8
5	120.9	120.9	124.1	120.9	120.7	120.9
6	122.9	122.9	125.0	122.9	122.9	122.9
7	111.7	111.7	112.8	111.7	111.3	111.7
7a	138.2	138.2	139.0	138.2	138.3	138.4
8	31.9	31.7	187.7	31.9	33.8	31.9
9	176.0	174.4		176.0	180.1	175.9
10		52.5				
	Xyl-*p*	Xyl-*p*	Xyl-*p*	Xyl-*p*	Xyl-*p*	Glc-*p*
1′	86.1	86.1	86.5	86.0	87.5	85.2
2′	79.0	79.0	78.9	77.3	73.7	81.2
3′	81.1	81.0	79.8	80.5	78.8	78.9
4′	70.9	70.9	70.7	77.6	71.1	71.2
5′	69.4	69.4	69.7	67.1	69.5	80.5
6′						62.6
	Glc-*p*	Glc-*p*	Glc-*p*	Glc-*p*		Glc-*p*
1″	105.0	105.0	104.5	104.9		104.9
2″	75.8	75.7	75.5	75.7		75.7
3″	77.7	77.7	77.7	77.8		77.7
4″	70.8	70.9	71.1	70.8		70.8
5″	77.4	77.4	77.6	77.2		77.4
6″	62.0	62.1	62.3	62.0		62.1
				Glc-*p*		
1‴				103.4		
2‴				74.6		
3‴				77.9		
4‴				71.5		
5‴				78.1		
6‴				62.6		

^a^ NMR data (*δ*) were measured at 150 MHz in CD_3_OD for **1**–**6**.

## Supplementary Materials

The following are available online: antitumor activities (IC_50_ μM, *n* = 3) of compounds **1**–**10** and 5-Fu (Table S1), UV, IR, HR-ESI-MS, 1D- and 2D-NMR spectra of compounds **1**–**6** (Figures S1–S42), cytotoxic activities of compounds **1**–**10** on PC12 cells at 10 μM (Figure S43).
