# Supplementary material for: New Indole Glycosides from Aesculus chinensis var. chekiangensis and Their Neuroprotective Activities
Source: Molecules. 2019 Nov 9;24(22):4063. doi: 10.3390/molecules24224063 (PMC6891264; doi:10.3390/molecules24224063)
Supplement: Supplementary file 1 [file molecules-24-04063-s001.pdf]

New indole glycosides from *Aesculus chinensis* Bge. var. *chekiangensis* (Hu et Fang) Fang and their neuroprotective activities

Nan Zhang [1,2], Shi-Jie Cao [2], Wei-Xing Huang [1,2], Pan Li [1,2], Ning Kang [1,2], Li-Qin Ding [2,*] and Feng Qiu [1,2,*]

[1] School of Chinese Materia Medica, Tianjin University of Traditional Chinese Medicine, Tianjin 301617, China; 13373750625@163.com (N.Z.); 1972074191@qq.com (W.-X.H.); 498560124@qq.com (P.L.); kangndd@163.com (N.K.); fengqiu20070118@163.com (F.Q.)

[2] Tianjin State Key Laboratory of Modern Chinese Medicine, Tianjin University of Traditional Chinese Medicine, Tianjin 301617, China; ruby70303@163.com (L.-Q.D.);

* Correspondence: ruby70303@163.com (L.-Q.D.); fengqiu20070118@163.com (F.Q.); Tel.: +86-22-59596223 (F.Q.)

Supporting Information

List of Supporting Information

S1. ^{1}H NMR Spectrum (600 MHZ, CD_3OD) of the new compound **1**
S2. ^{13}C NMR Spectrum (150 MHZ, CD_3OD) of the new compound **1**
S3. HSQC Spectrum of the new compound **1**
S4. HMBC Spectrum of the new compound **1**
S5. HRESIMS spectrum of the new compound **1**
S6. UV spectrum of the new compound **1**
S7. IR spectrum of the new compound **1**
S8. ^{1}H NMR Spectrum (600 MHZ, CD_3OD) of the new compound **2**
S9. ^{13}C NMR Spectrum (150 MHZ, CD_3OD) of the new compound **2**
S10. HSQC Spectrum of the new compound **2**
S11. HMBC Spectrum of the new compound **2**
S12. HRESIMS spectrum of the new compound **2**
S13. UV spectrum of the new compound **2**
S14. IR spectrum of the new compound **2**
S15. ^{1}H NMR Spectrum (600 MHZ, CD_3OD) of the new compound **3**
S16. ^{13}C NMR Spectrum (150 MHZ, CD_3OD) of the new compound **3**
S17. HSQC Spectrum of the new compound **3**
S18. HMBC Spectrum of the new compound **3**
S19. HRESIMS spectrum of the new compound **3**
S20. UV spectrum of the new compound **3**
S21. IR spectrum of the new compound **3**
S22. ^{1}H NMR Spectrum (600 MHZ, CD_3OD) of the new compound **4**
S23. ^{13}C NMR Spectrum (150 MHZ, CD_3OD) of the new compound **4**
S24. HSQC Spectrum of the new compound **4**
S25. HMBC Spectrum of the new compound **4**
S26. HRESIMS spectrum of the new compound **4**
S27. UV spectrum of the new compound **4**
S28. IR spectrum of the new compound **4**
S29. ^{1}H NMR Spectrum (600 MHZ, CD_3OD) of the new compound **5**
S30. ^{13}C NMR Spectrum (150 MHZ, CD_3OD) of the new compound **5**
S31. HSQC Spectrum of the new compound **5**
S32. HMBC Spectrum of the new compound **5**
S33. HRESIMS spectrum of the new compound **5**
S34. UV spectrum of the new compound **5**
S35. IR spectrum of the new compound **5**
S36. ^{1}H NMR Spectrum (600 MHZ, CD_3OD) of the new compound **6**
S37. ^{13}C NMR Spectrum (150 MHZ, CD_3OD) of the new compound **6**
S38. HSQC Spectrum of the new compound **6**
S39. HMBC Spectrum of the new compound **6**
S40. HRESIMS spectrum of the new compound **6**
S41. UV spectrum of the new compound **6**
S42. IR spectrum of the new compound **6**

S43. Cytotoxic activities of compounds **1-10** on PC12 cells at 10 Mm

Table S1. Antitumor activities (IC_{50} μM, n = 3) of compounds **1-10** and 5-Fu. The data are expressed as means ± SEM.

S1. ^{1}H NMR Spectrum (600 MHZ, CD_3OD) of the new compound **1**

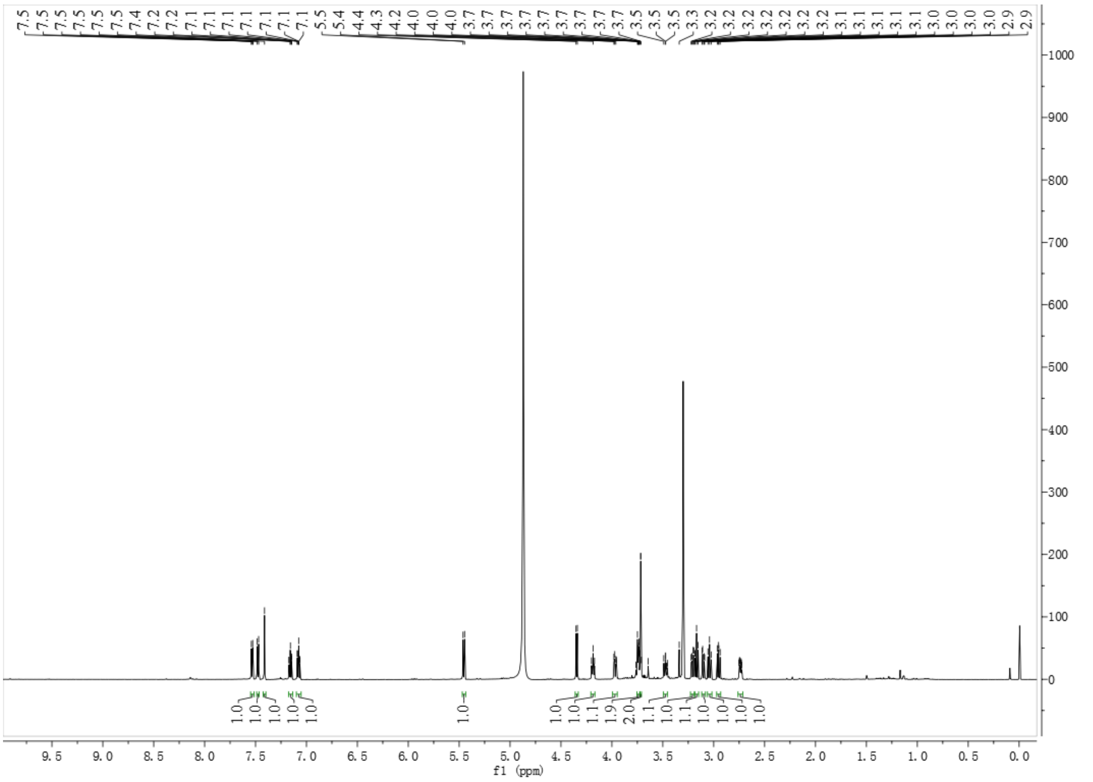

S2. ^{13}C NMR Spectrum (150 MHZ, CD_3OD) of the new compound **1**

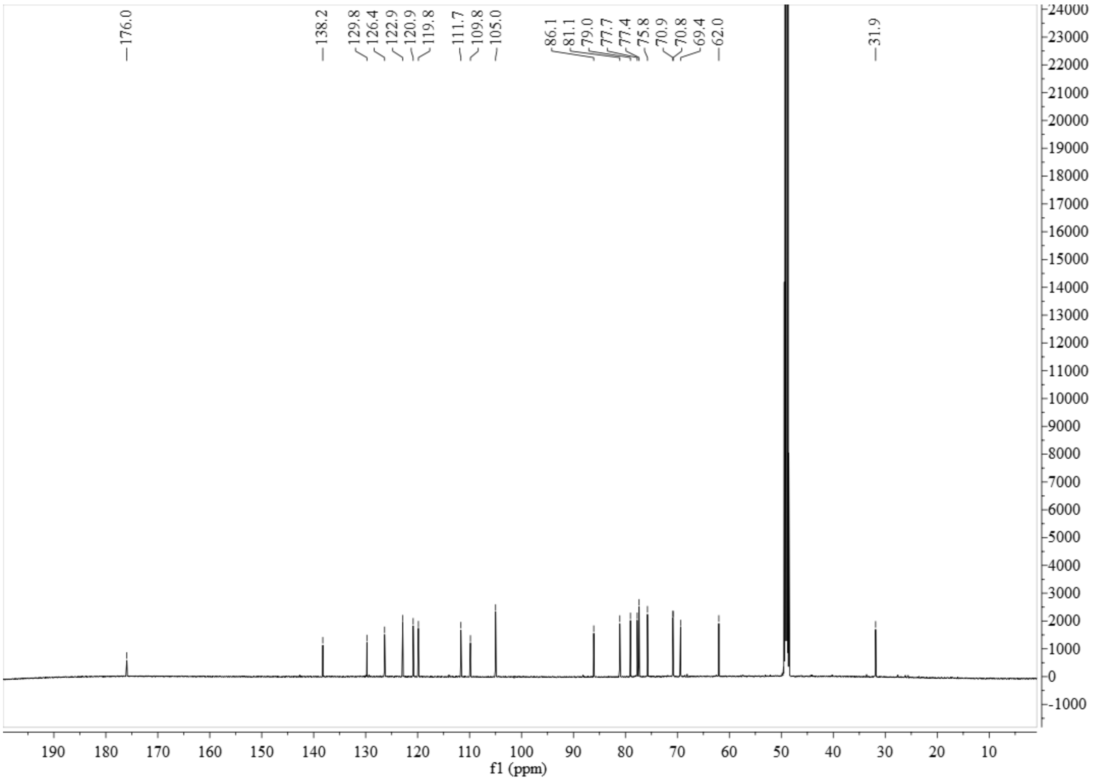

S3. HSQC Spectrum of the new compound **1**

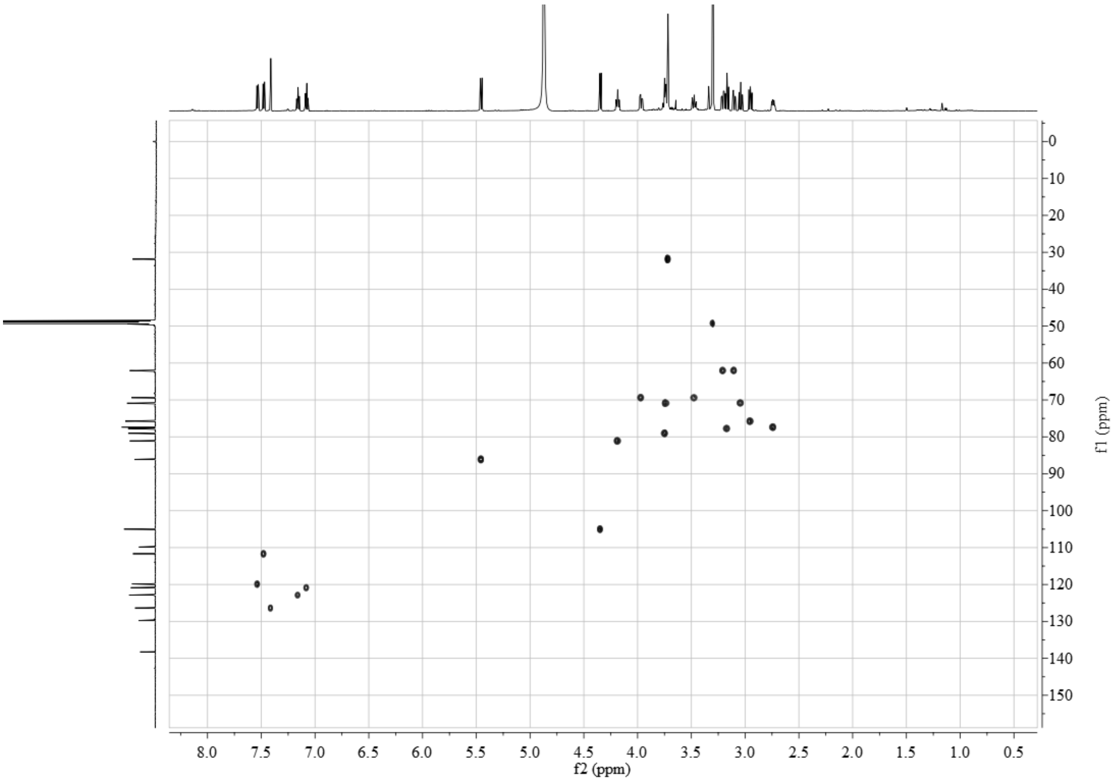

S4. HMBC Spectrum of the new compound **1**

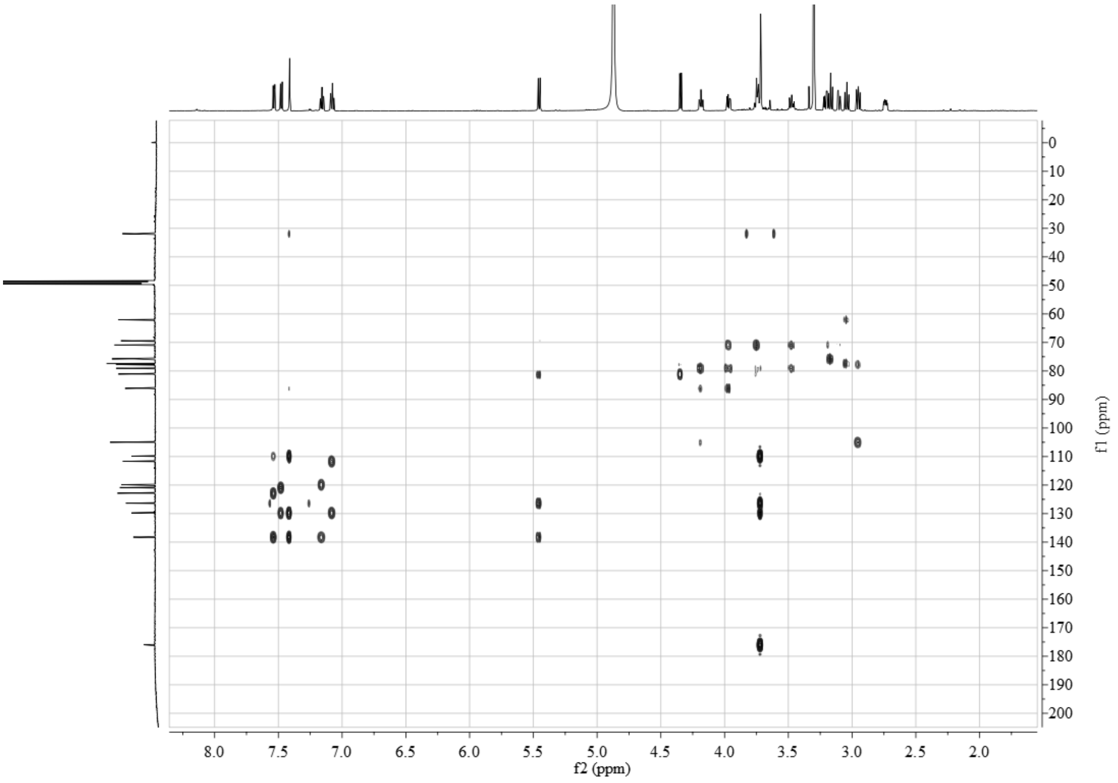

S5. HRESIMS spectrum of the new compound **1**

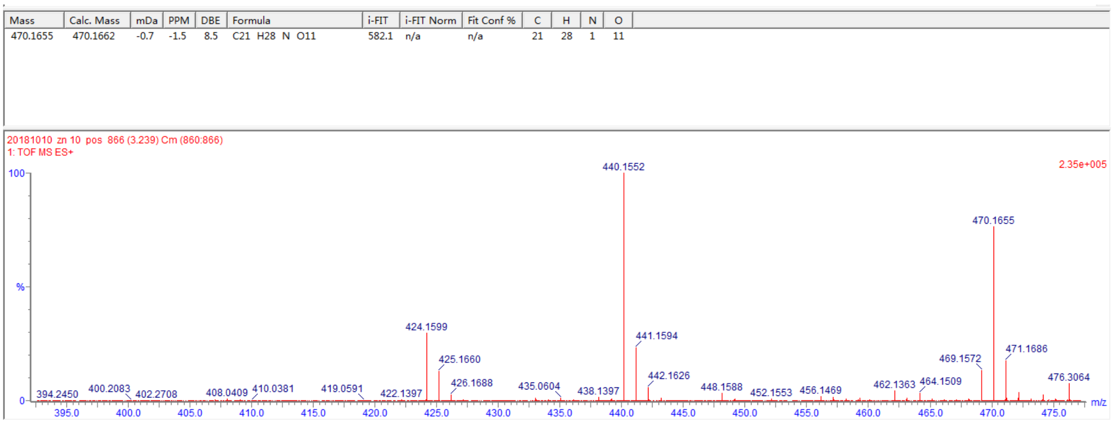

Mass	Calc. Mass	mDa	PPM	DBE	Formula	i-FIT	i-FIT Norm	Fit Conf %	C	H	N	O
470.1655	470.1662	-0.7	-1.5	8.5	C21 H28 N O11	582.1	n/a	n/a	21	28	1	11

S6. UV spectrum of the new compound **1**

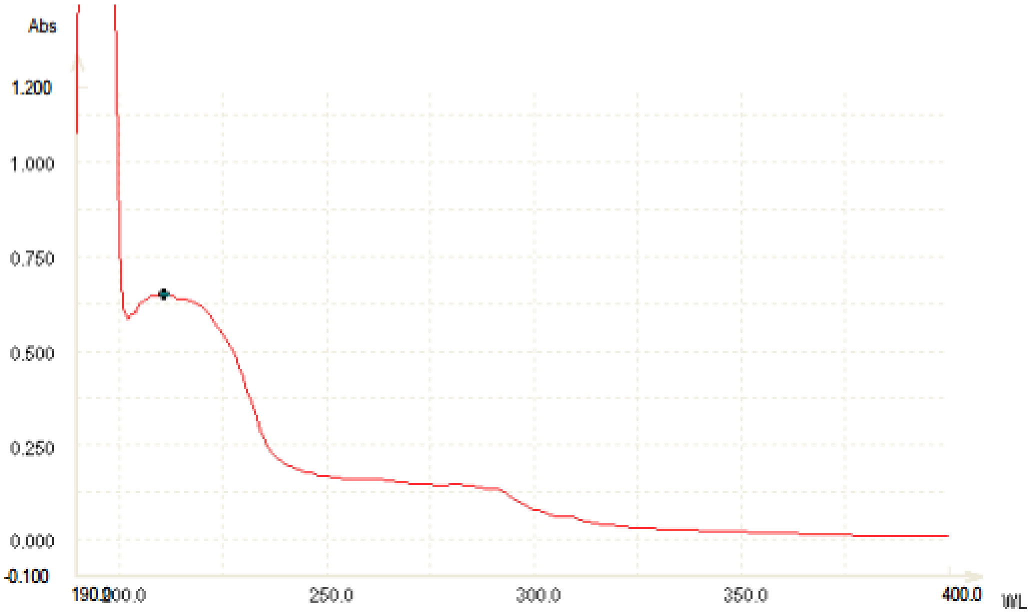

S7. IR spectrum of the new compound **1**

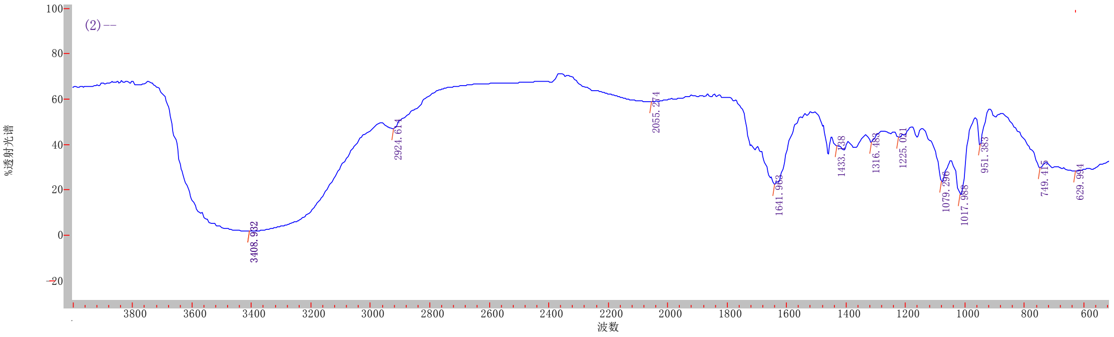

S8. ^{1}H NMR Spectrum (600 MHZ, CD_3OD) of the new compound **2**

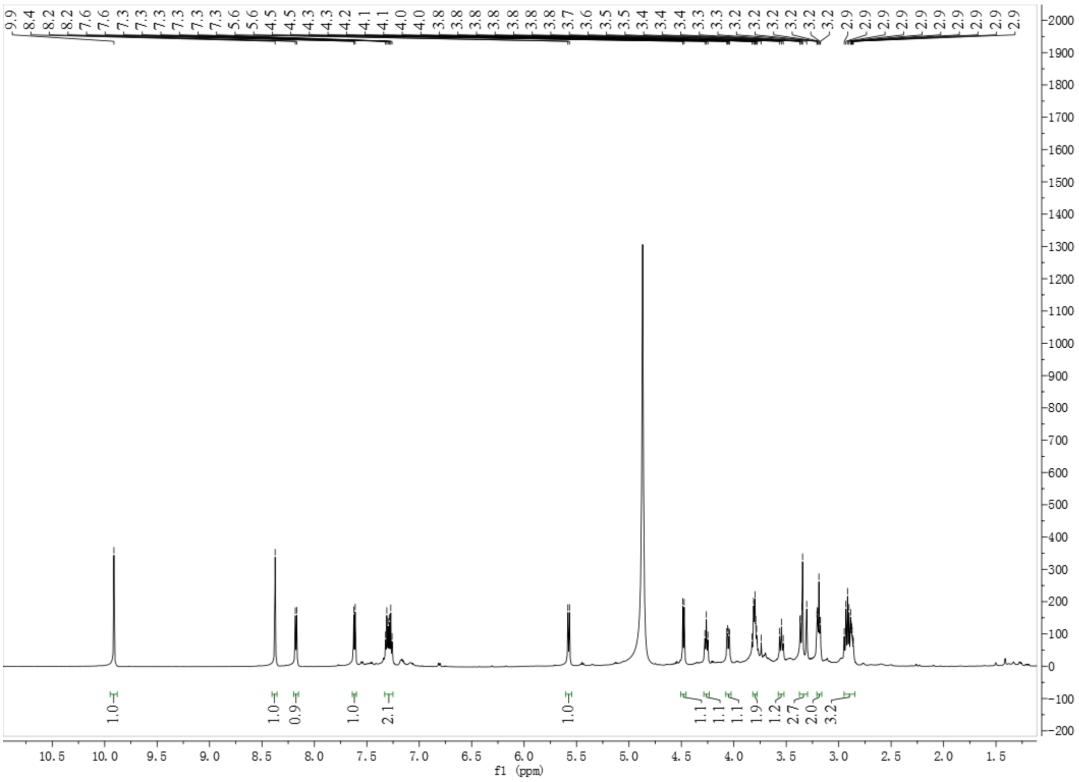

S9. ^{13}C NMR Spectrum (150 MHZ, CD_3OD) of the new compound **2**

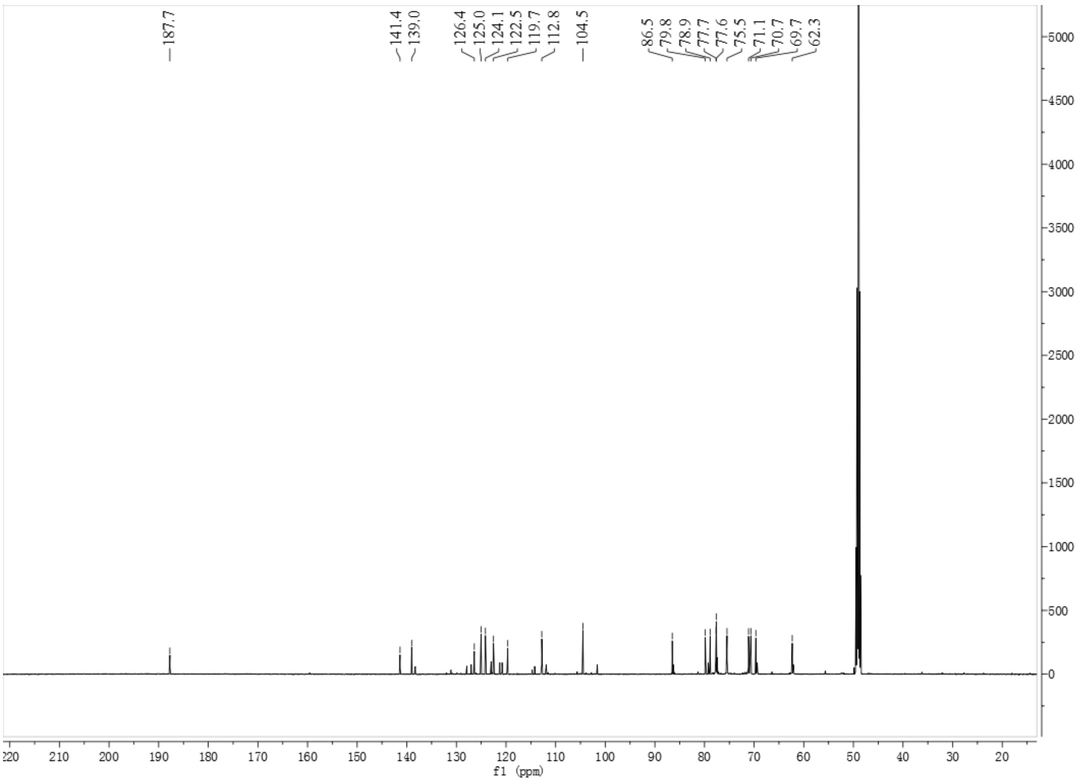

S10. HSQC Spectrum of the new compound **2**

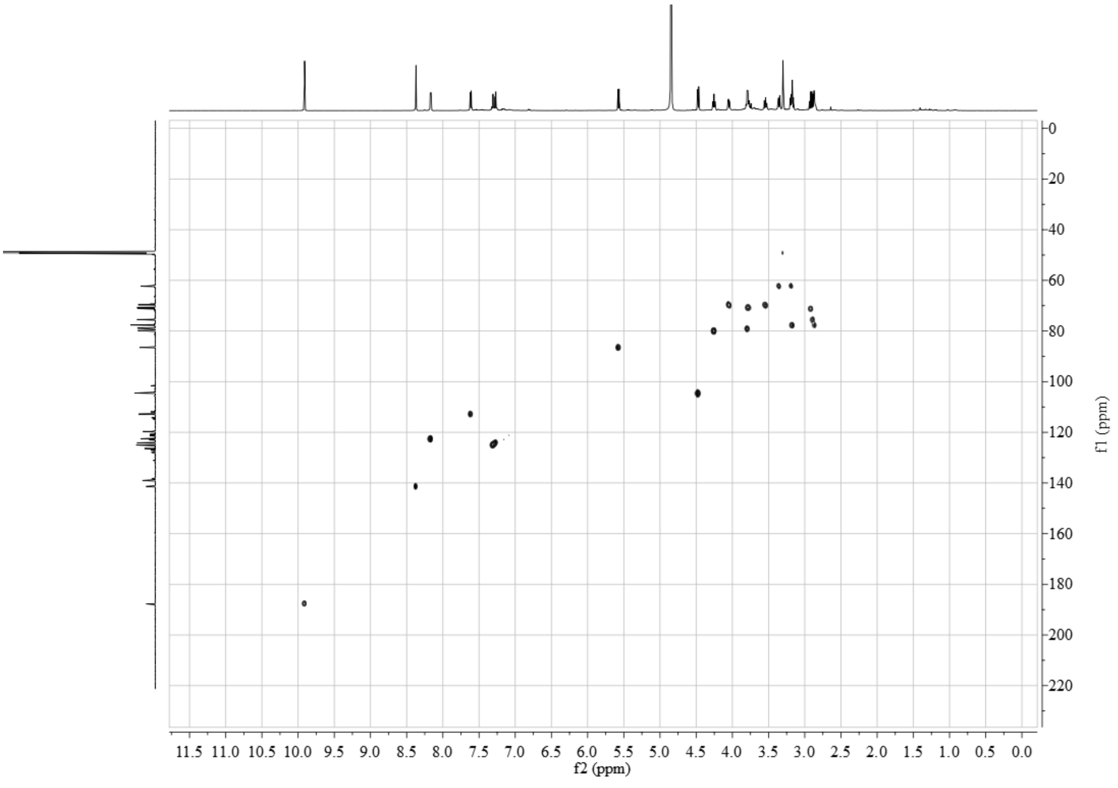

S11. HMBC Spectrum of the new compound **2**

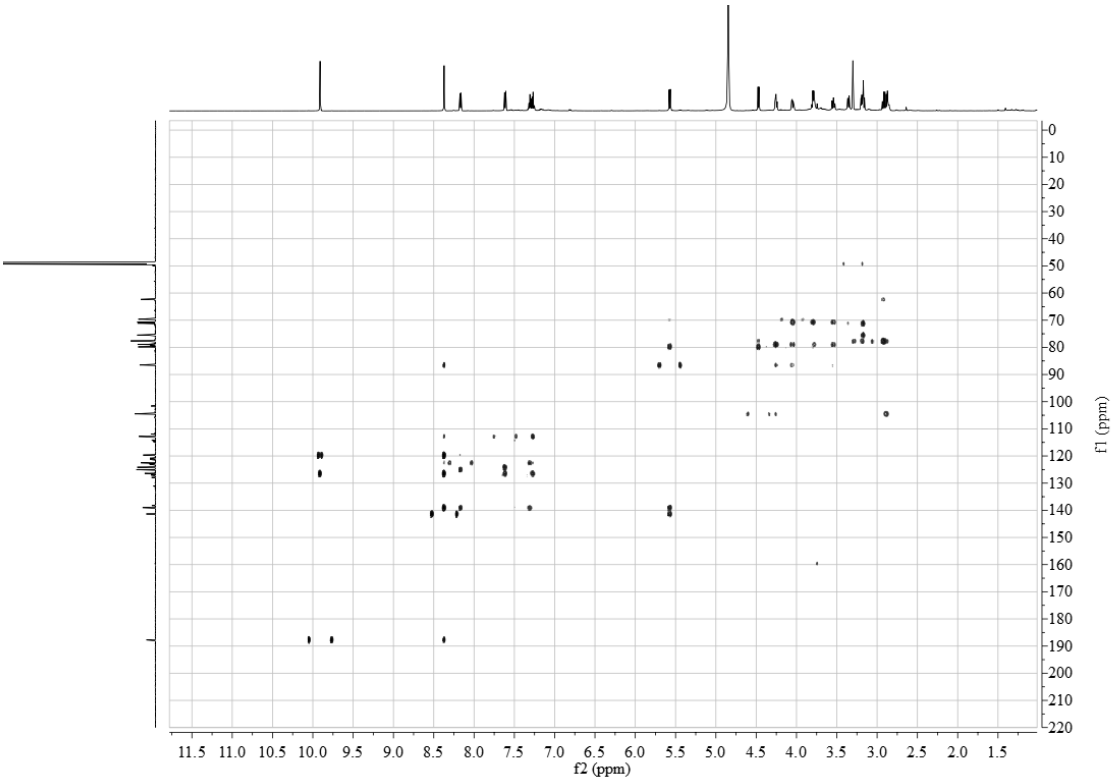

S12. HRESIMS spectrum of the new compound **2**

Mass	Calc. Mass	mDa	PPM	DBE	Formula	i-FIT	i-FIT Norm	Fit Conf %	C	H	N	O
440.1549	440.1557	-0.8	-1.8	8.5	C20 H26 N O10	473.9	n/a	n/a	20	26	1	10

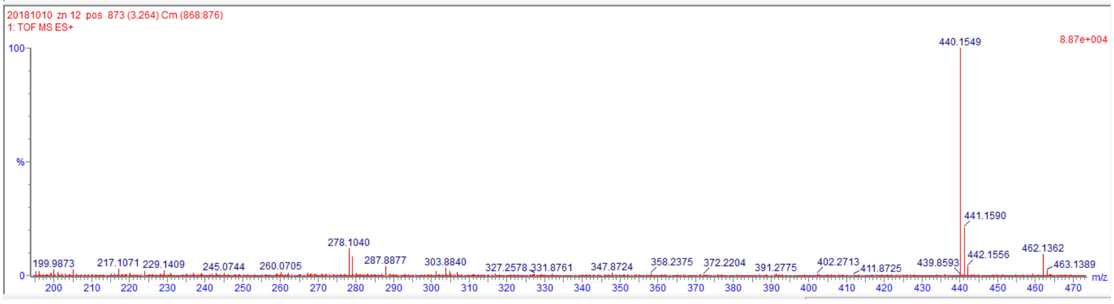

S13. UV spectrum of the new compound **2**

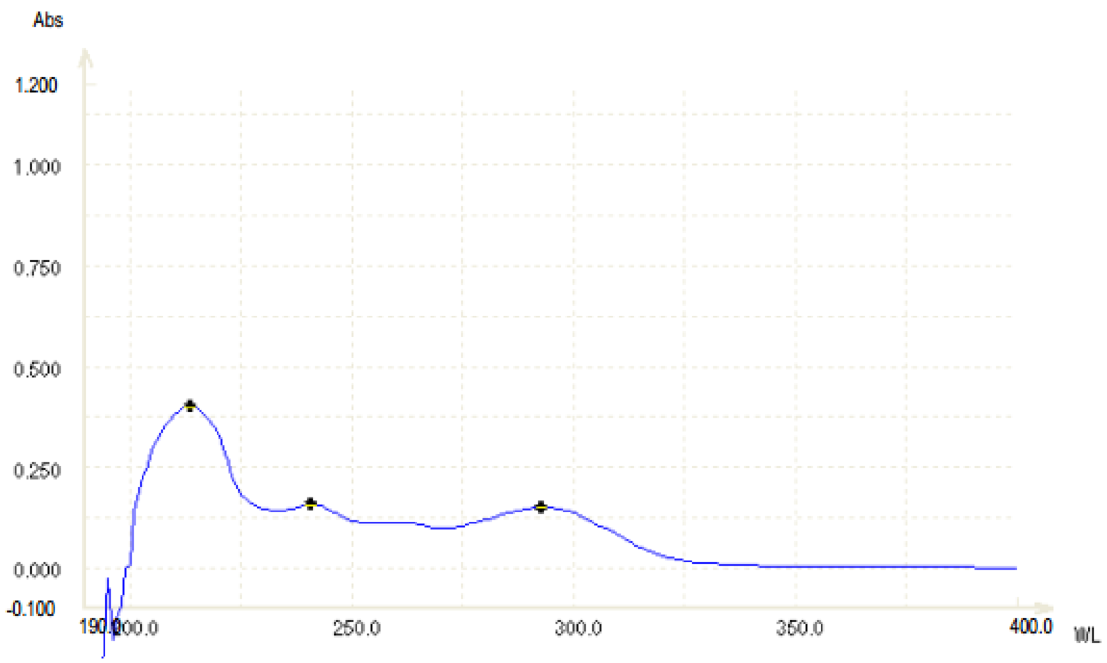

S14. IR spectrum of the new compound **2**

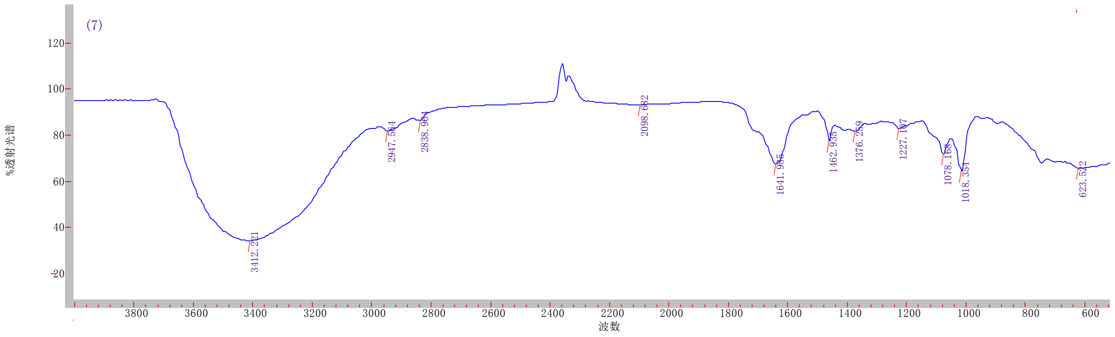

S15. ^{1}H NMR Spectrum (600 MHZ, CD_3OD) of the new compound **3**

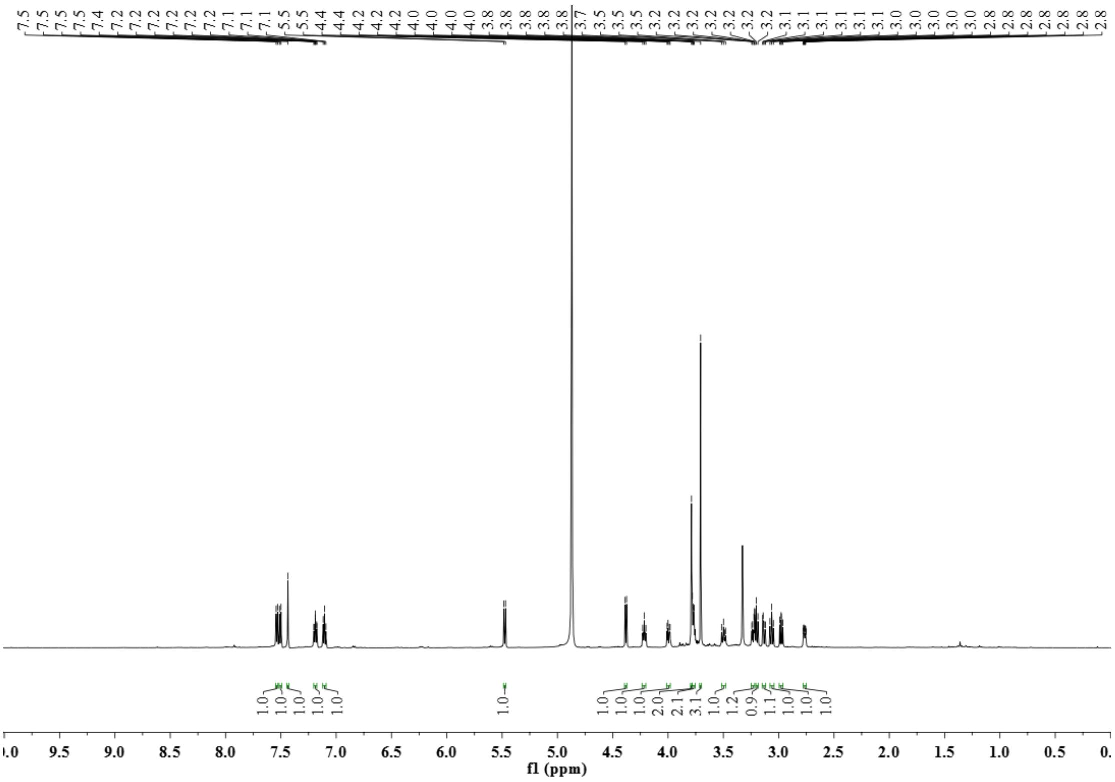

S16. ^{13}C NMR Spectrum (150 MHZ, CD_3OD) of the new compound **3**

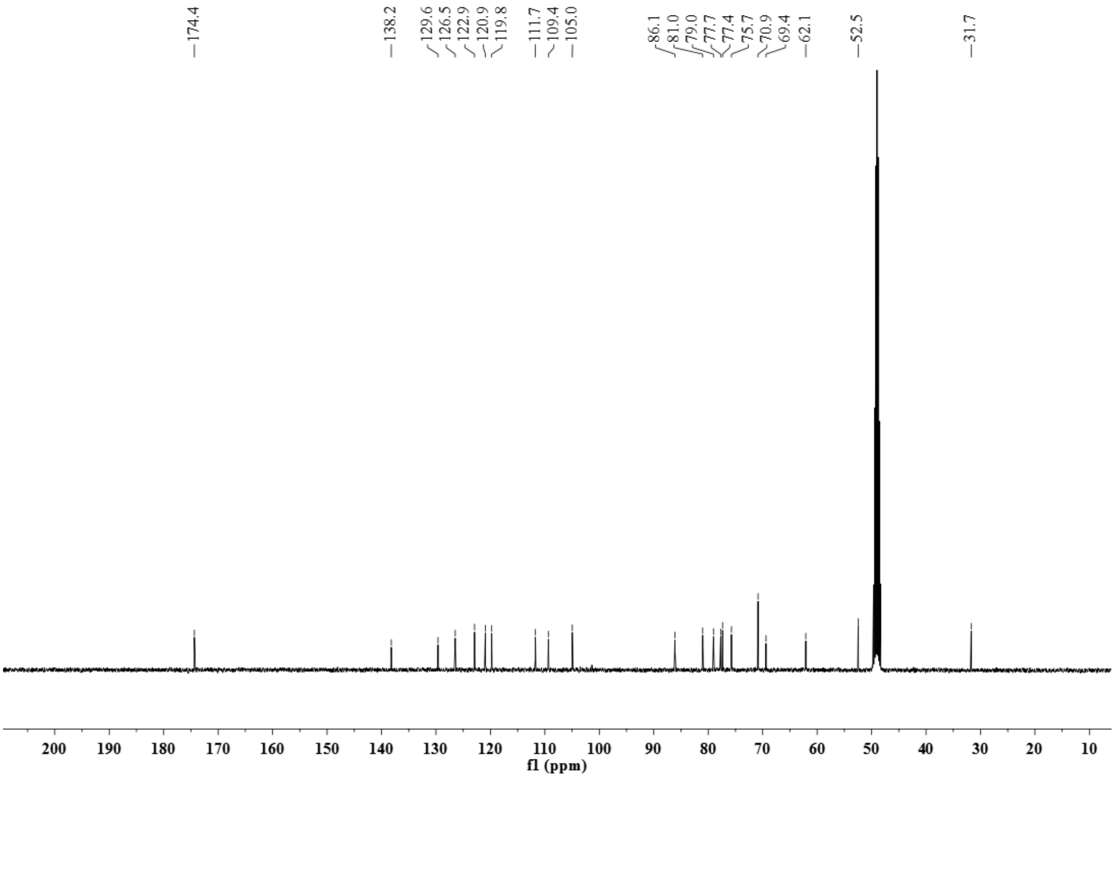

S17. HSQC Spectrum of the new compound **3**

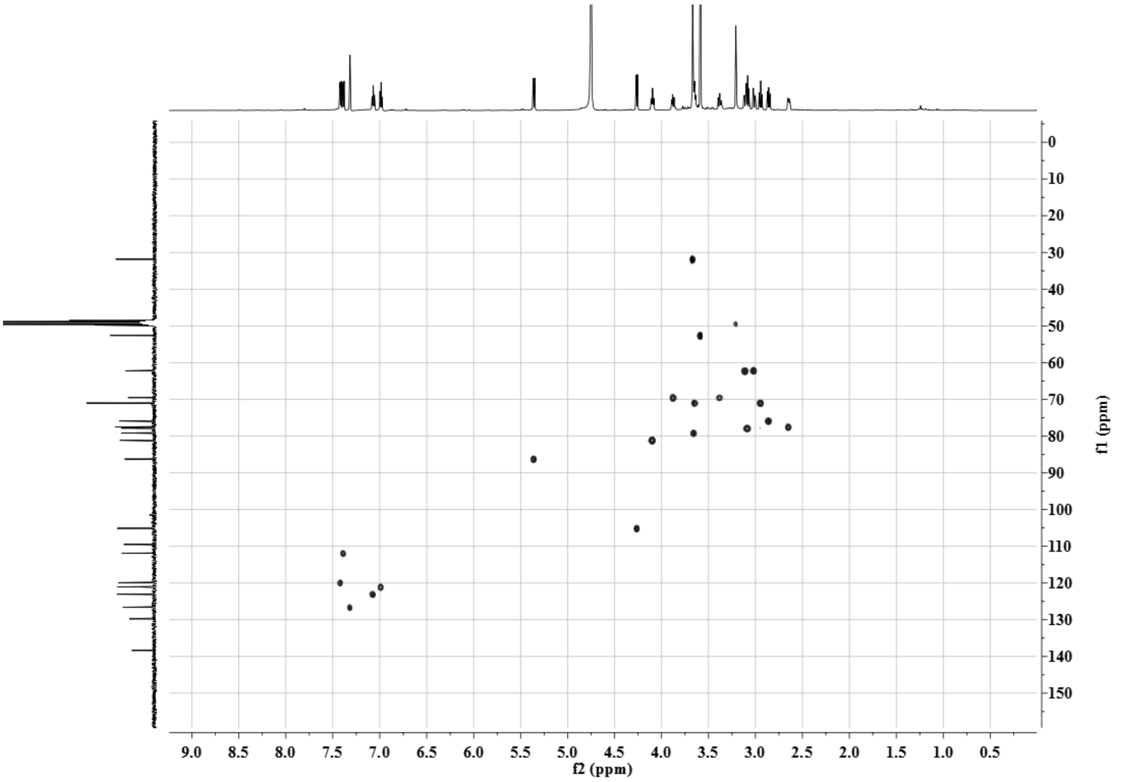

S18. HMBC Spectrum of the new compound **3**

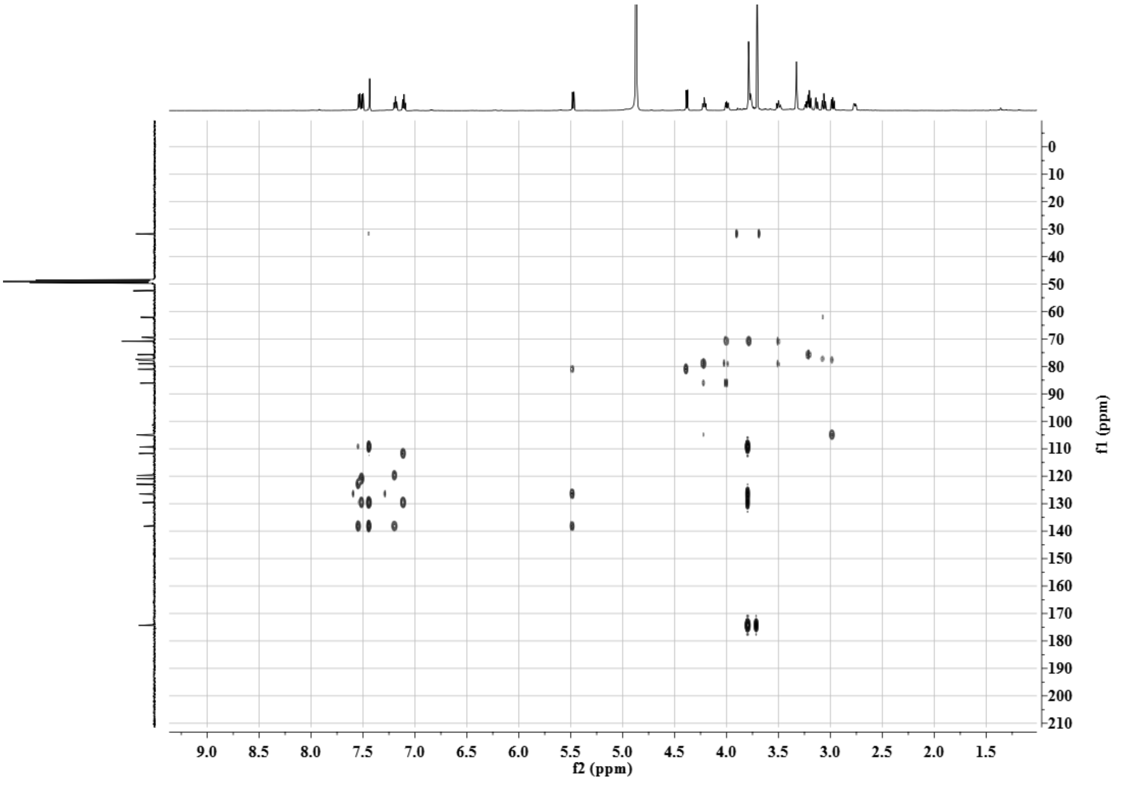

S19. HRESIMS spectrum of the new compound **3**

Mass	Calc. Mass	mDa	PPM	DBE	Formula	i-FIT	i-FIT Norm	Fit Conf %	C	H	N	O
484.1811	484.1819	-0.8	-1.7	8.5	C22 H30 N O11	598.4	n/a	n/a	22	30	1	11

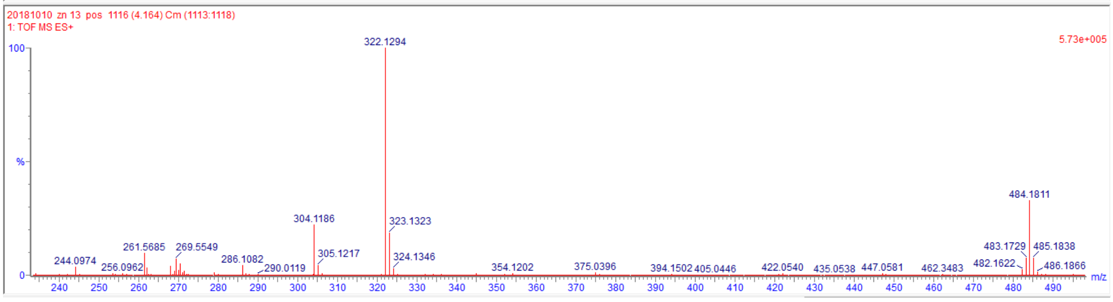

S20. UV spectrum of the new compound **3**

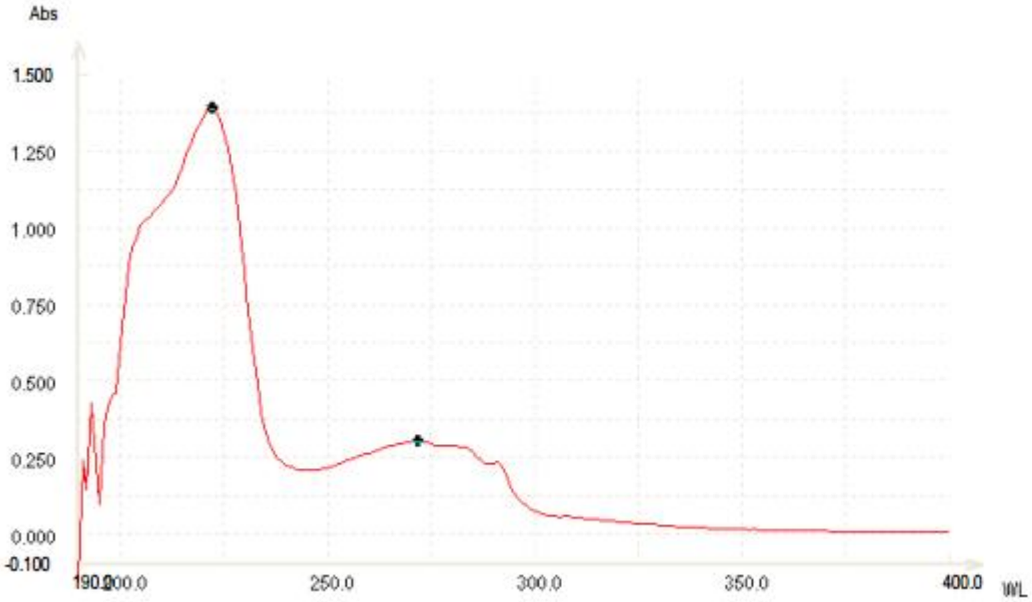

S21. IR spectrum of the new compound **3**

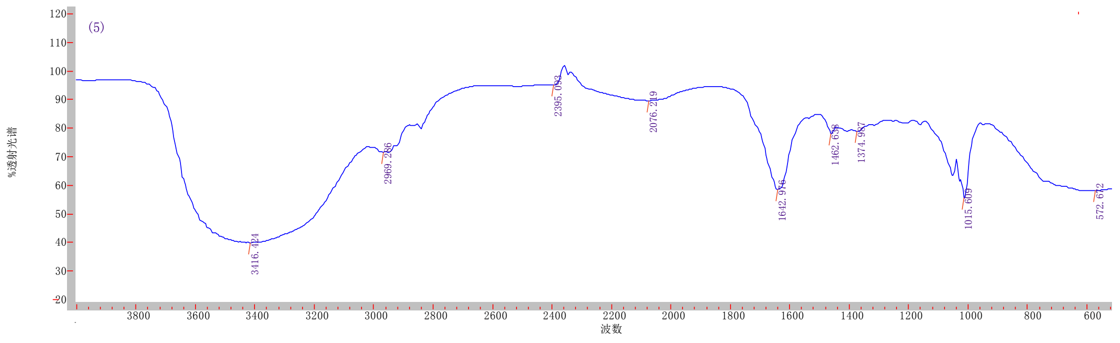

S22. ^{1}H NMR Spectrum (600 MHZ, CD_3OD) of the new compound **4**

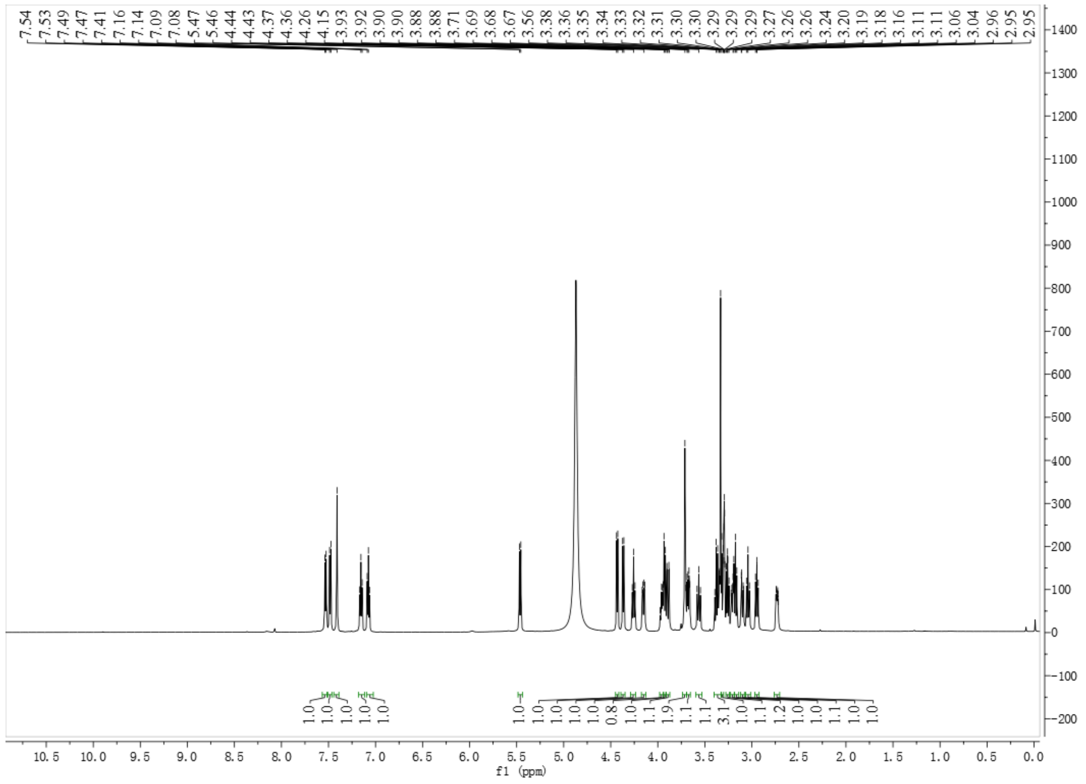

S23. ^{13}C NMR Spectrum (150 MHZ, CD_3OD) of the new compound **4**

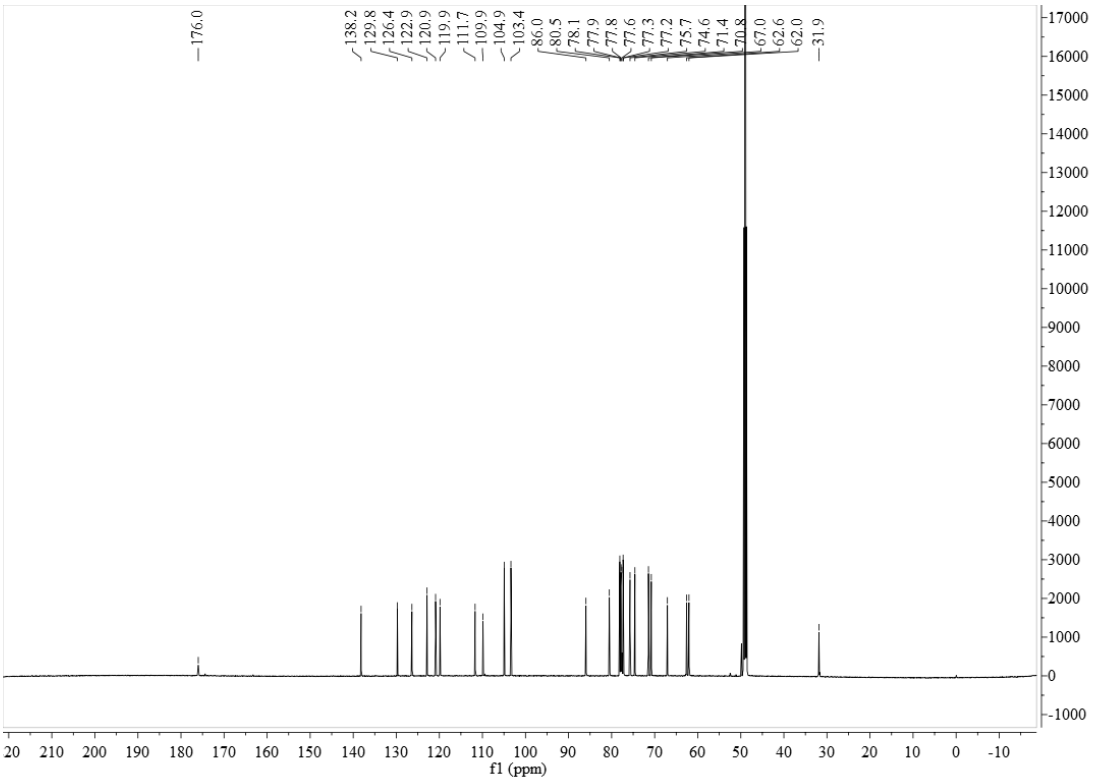

S24. HSQC Spectrum of the new compound **4**

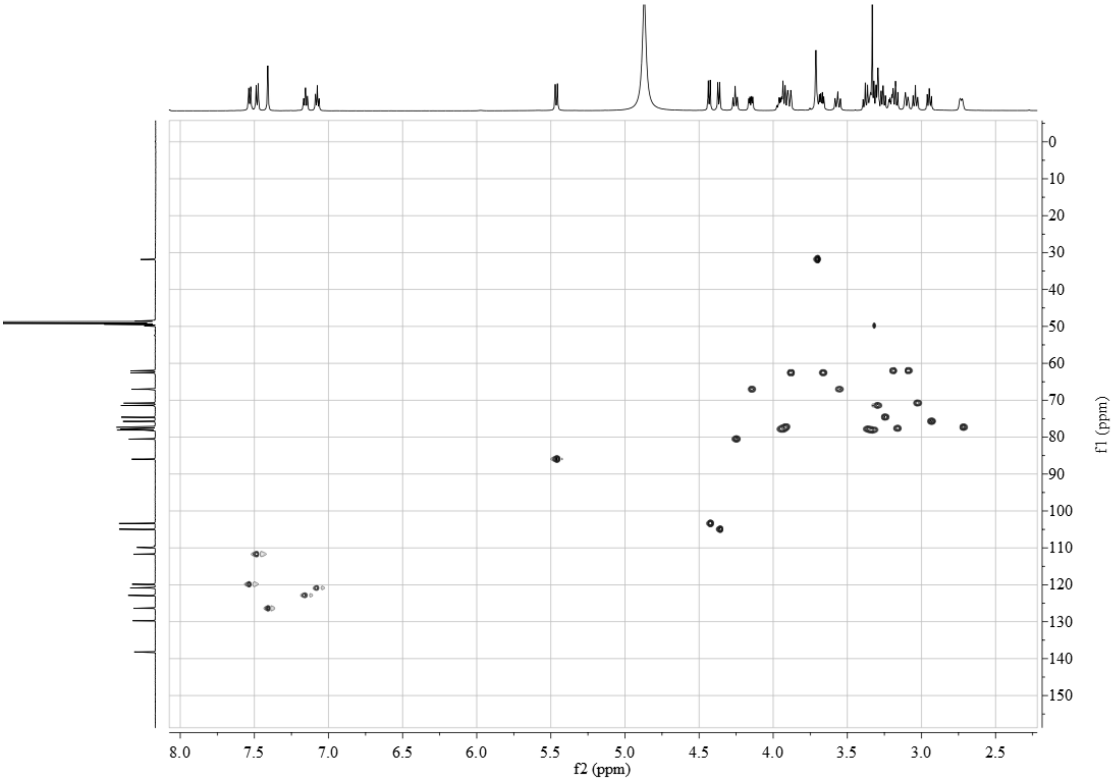

S25. HMBC Spectrum of the new compound **4**

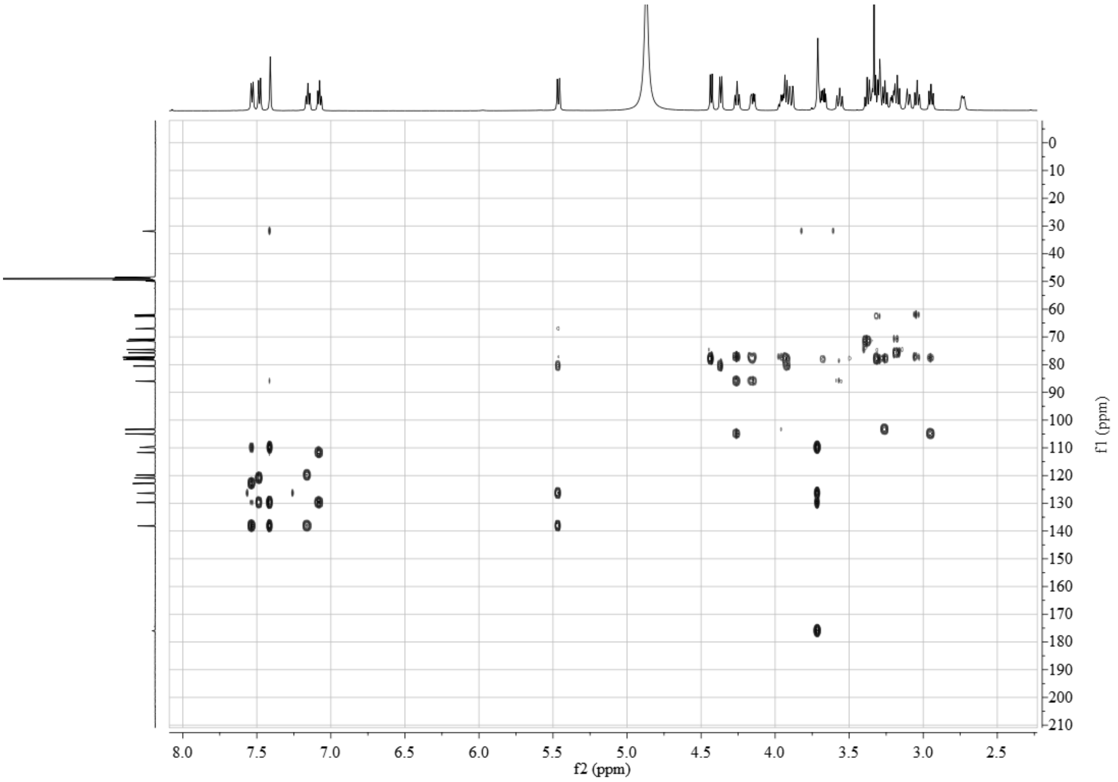

S26. HRESIMS spectrum of the new compound **4**

Mass	Calc. Mass	mDa	PPM	DBE	Formula	i-FIT	i-FIT Norm	Fit Conf %	C	H	N	O
632.2150	632.2191	-4.1	-6.5	9.5	C27 H38 N O16	187.8	n/a	n/a	27	38	1	16

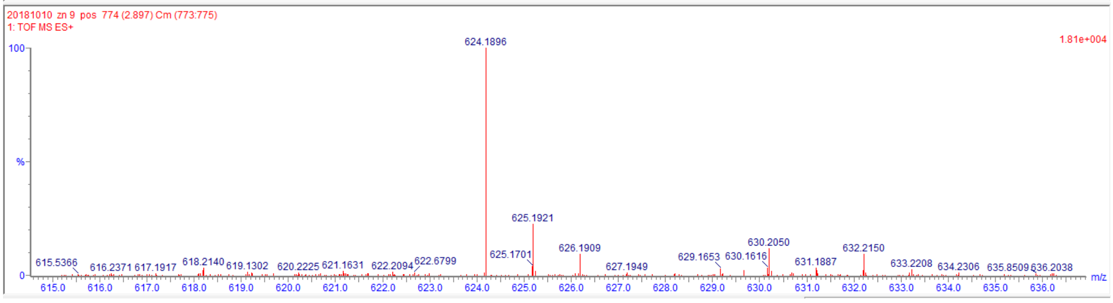

S27. UV spectrum of the new compound **4**

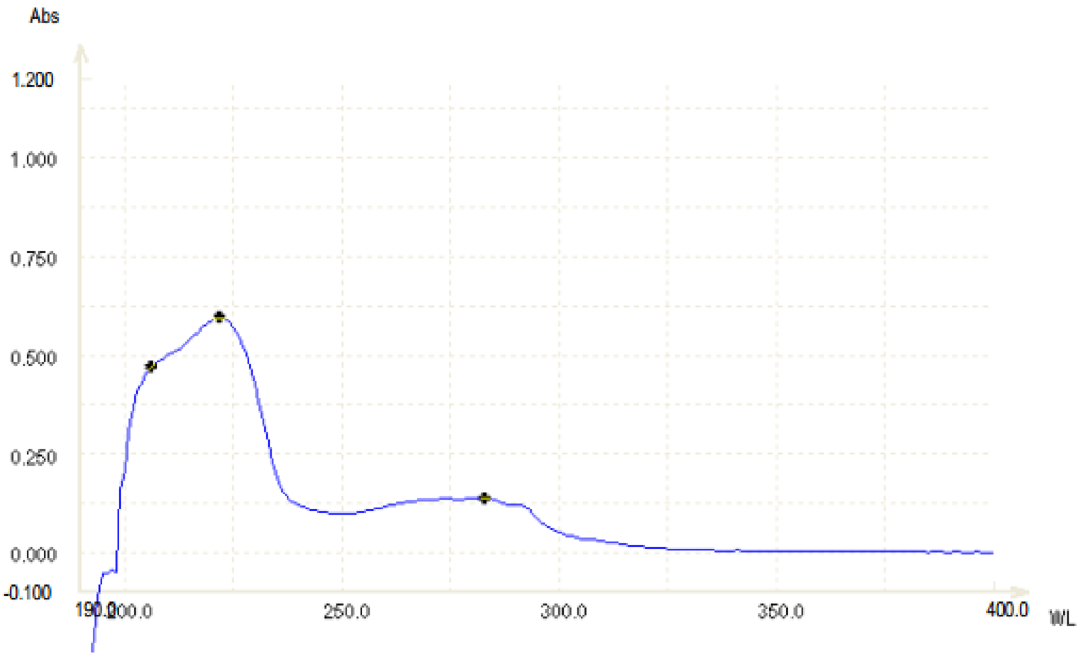

S28. IR spectrum of the new compound **4**

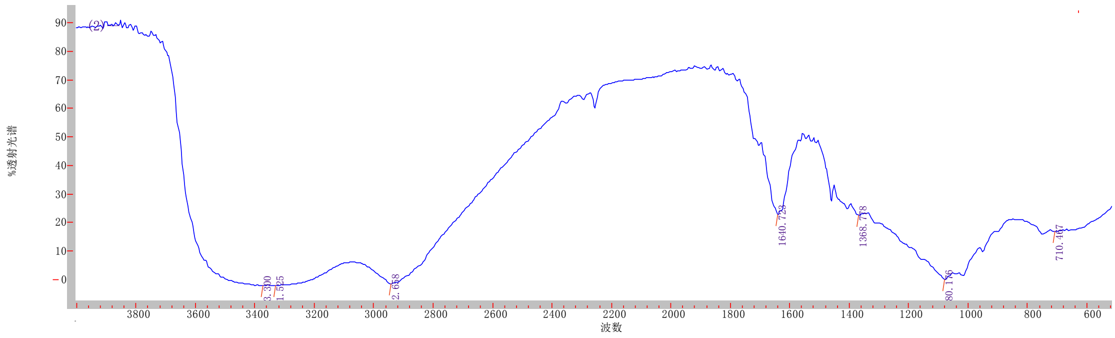

S29. ^{1}H NMR Spectrum (600 MHZ, CD_3OD) of the new compound **5**

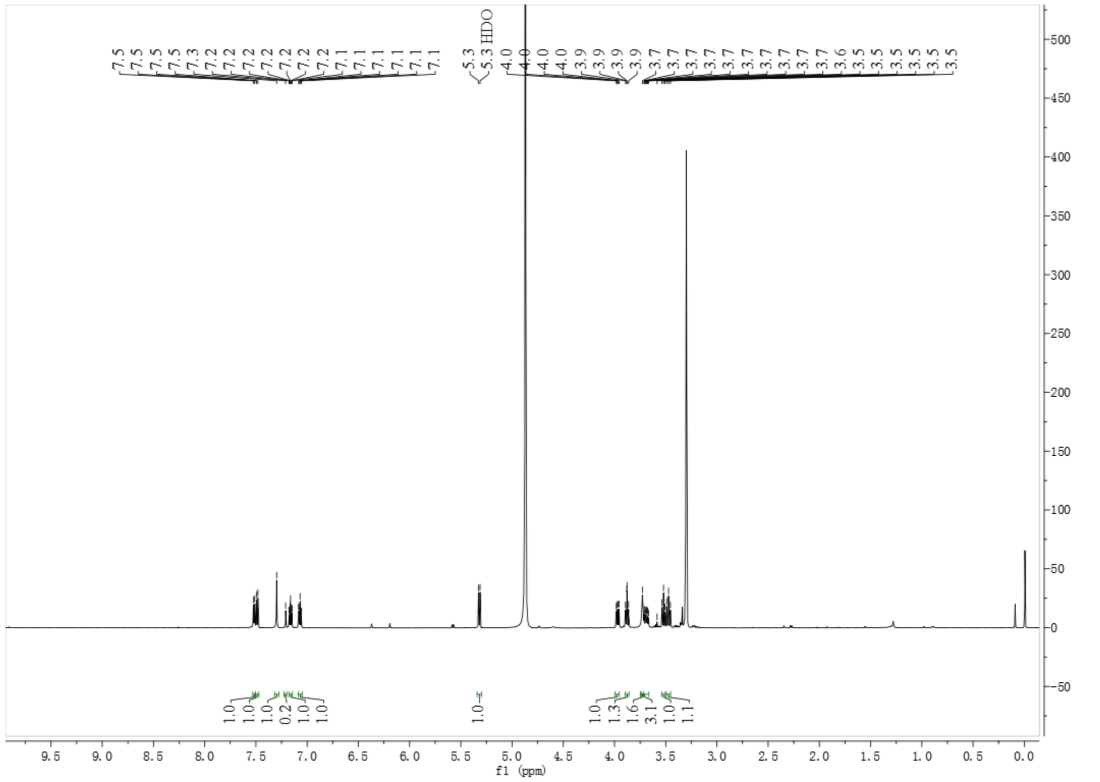

S30. ^{13}C NMR Spectrum (150 MHZ, CD_3OD) of the new compound **5**

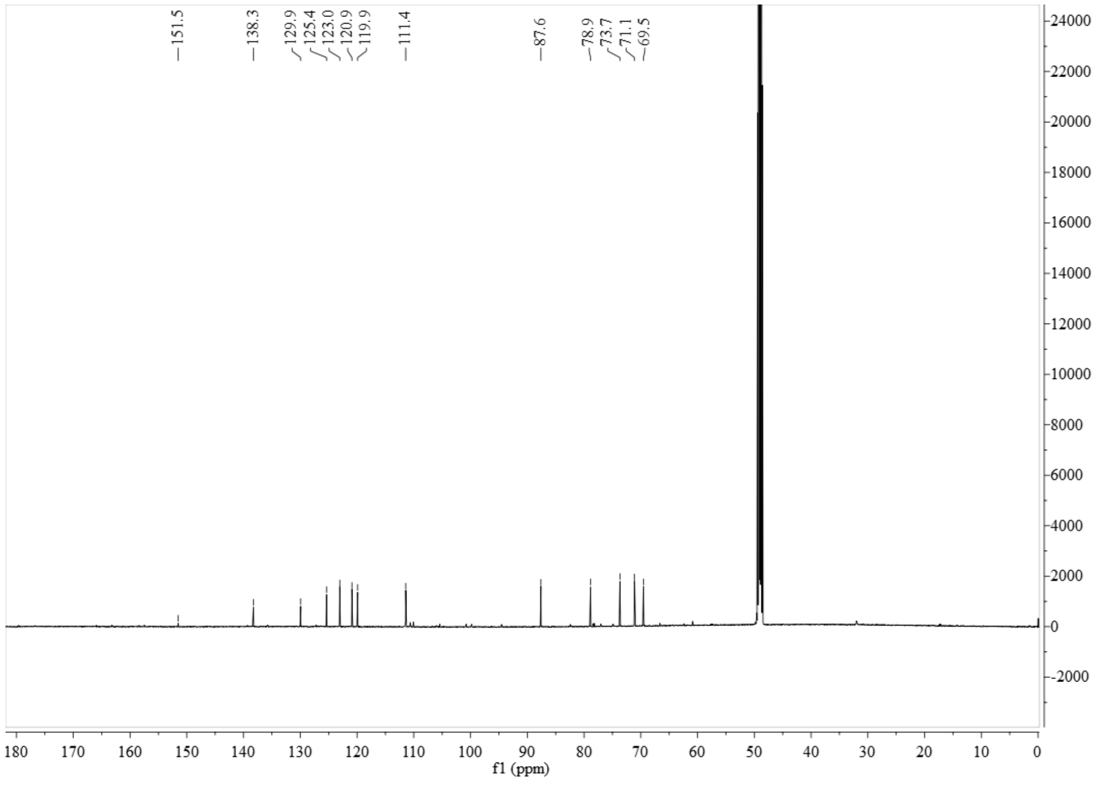

S31. HSQC Spectrum of the new compound **5**

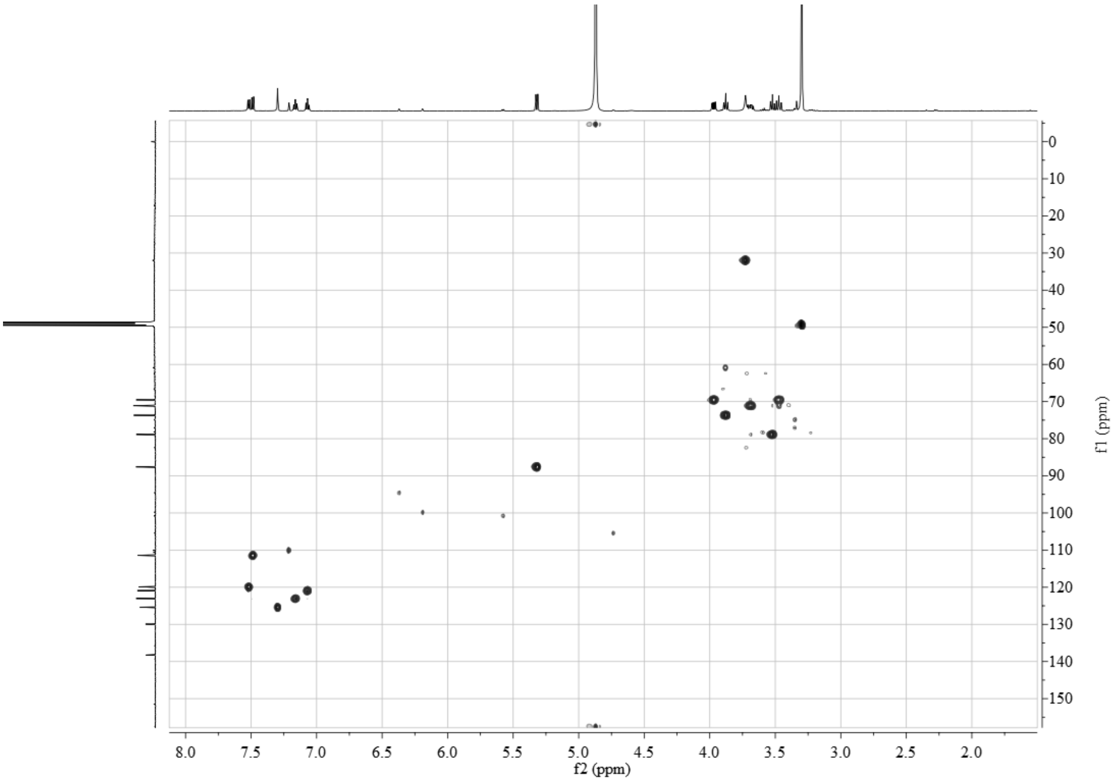

S32. HMBC Spectrum of the new compound **5**

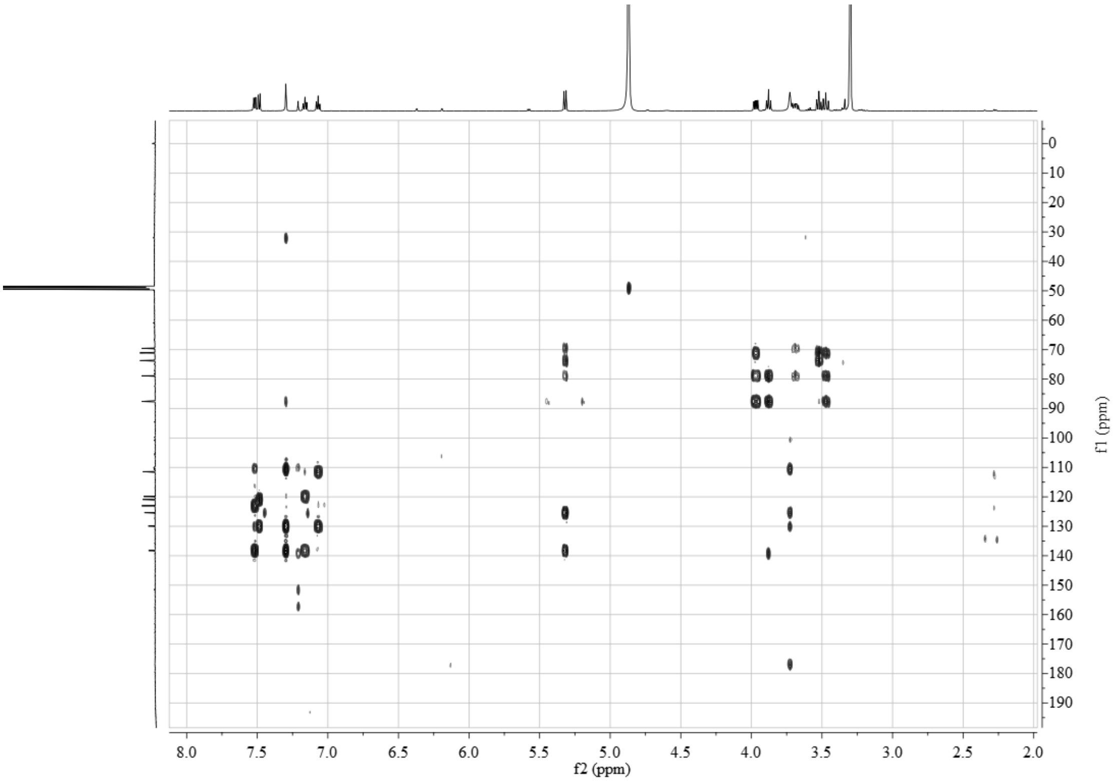

S33. HRESIMS spectrum of the new compound **5**

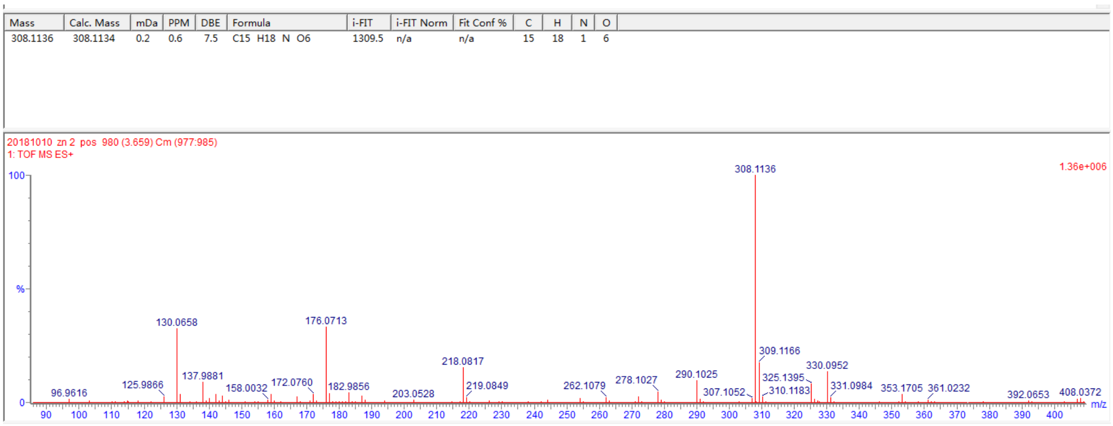

Mass	Calc. Mass	mDa	PPM	DBE	Formula	i-FIT	i-FIT Norm	Fit Conf %	C	H	N	O
308.1136	308.1134	0.2	0.6	7.5	C15 H18 N O6	1309.5	n/a	n/a	15	18	1	6

S34. UV spectrum of the new compound **5**

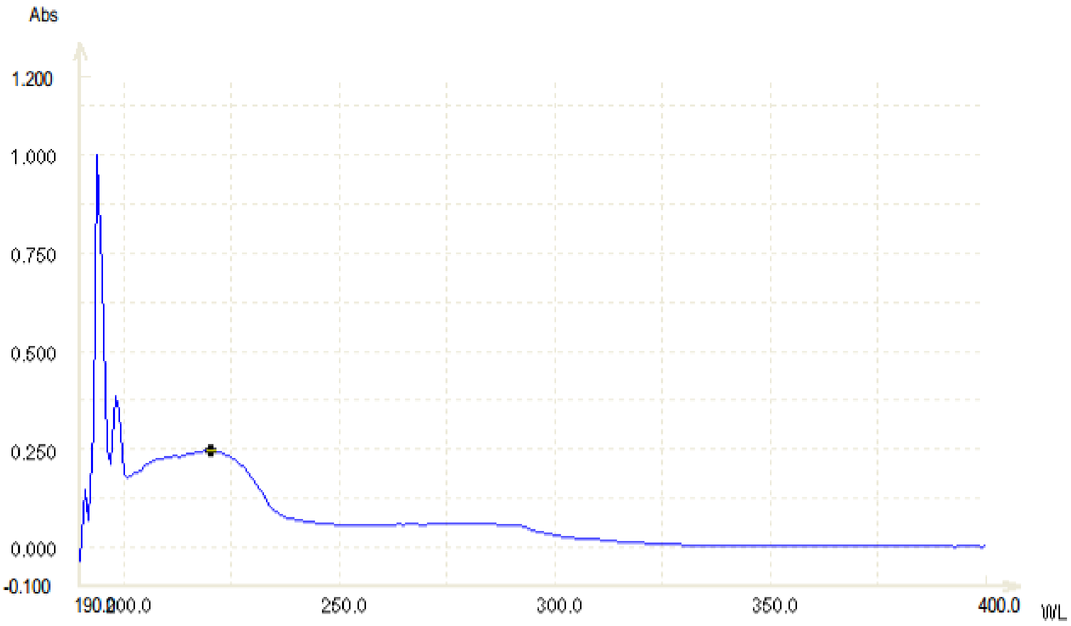

S35. IR spectrum of the new compound **5**

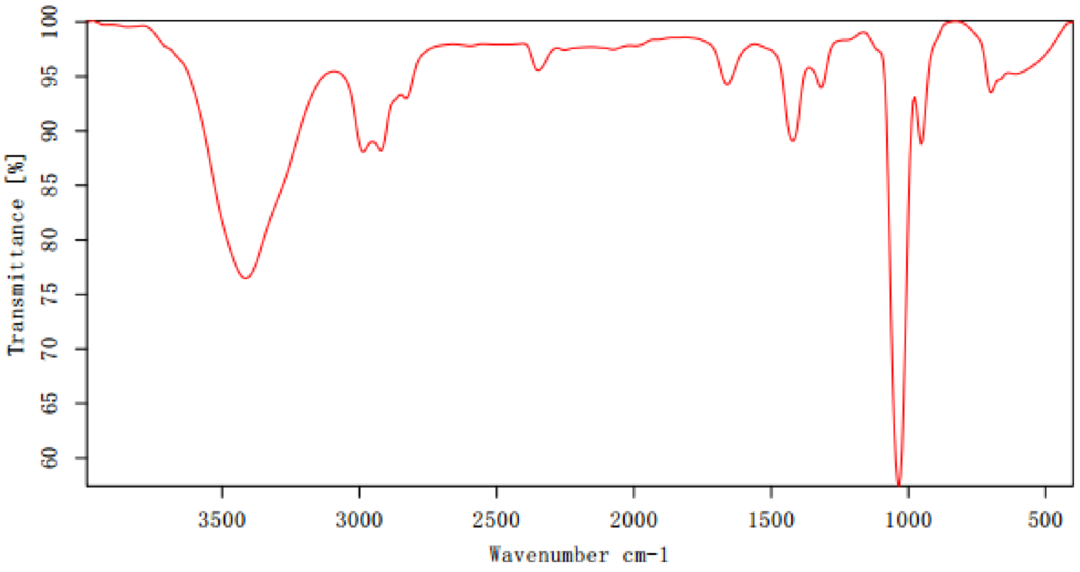

S36. ^{1}H NMR Spectrum (600 MHZ, CD_3OD) of the new compound **6**

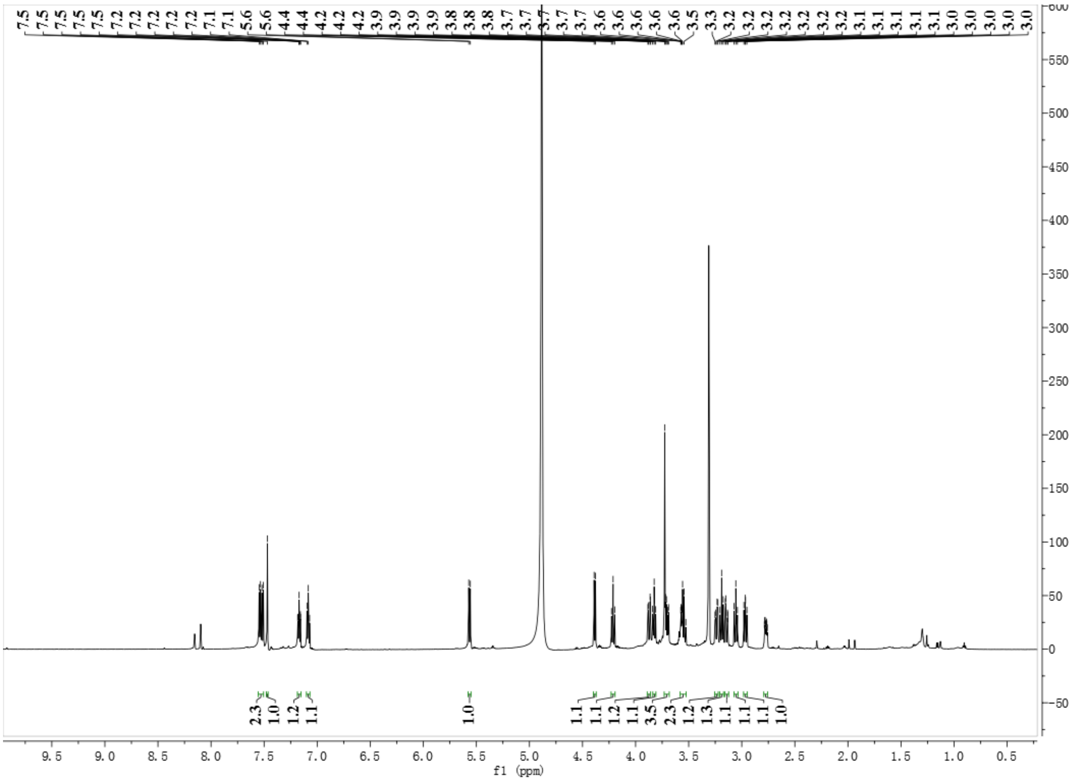

S37. ^{13}C NMR Spectrum (150 MHZ, CD_3OD) of the new compound **6**

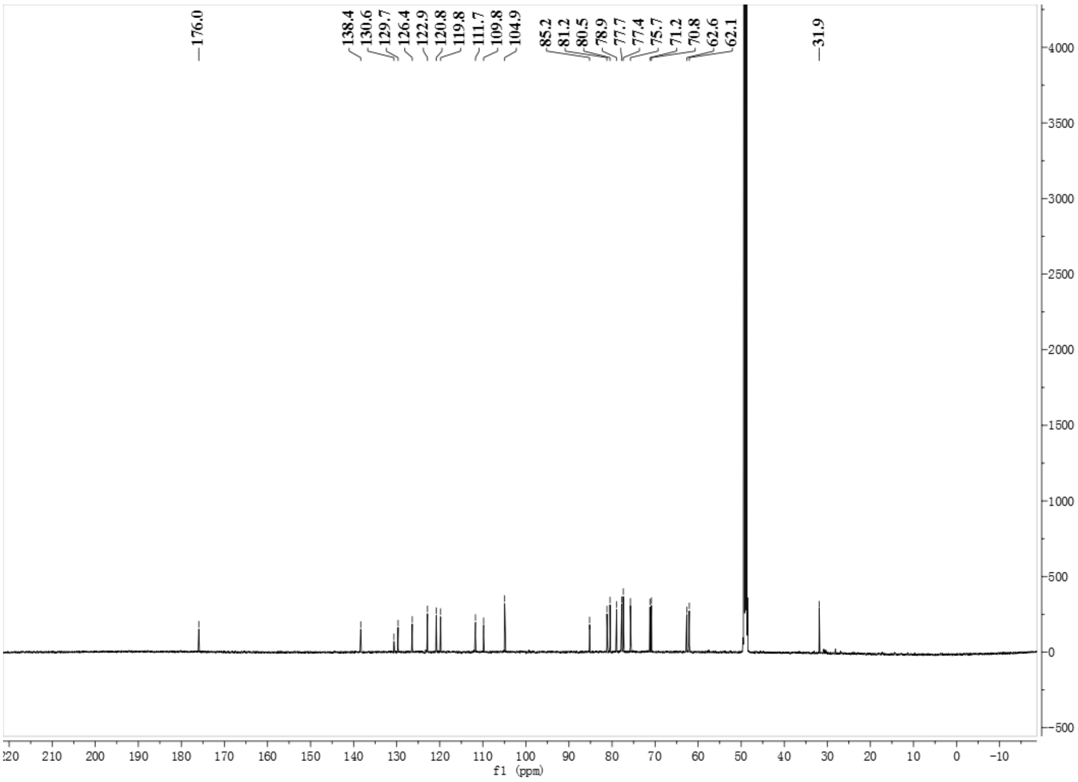

S38. HSQC Spectrum of the new compound **6**

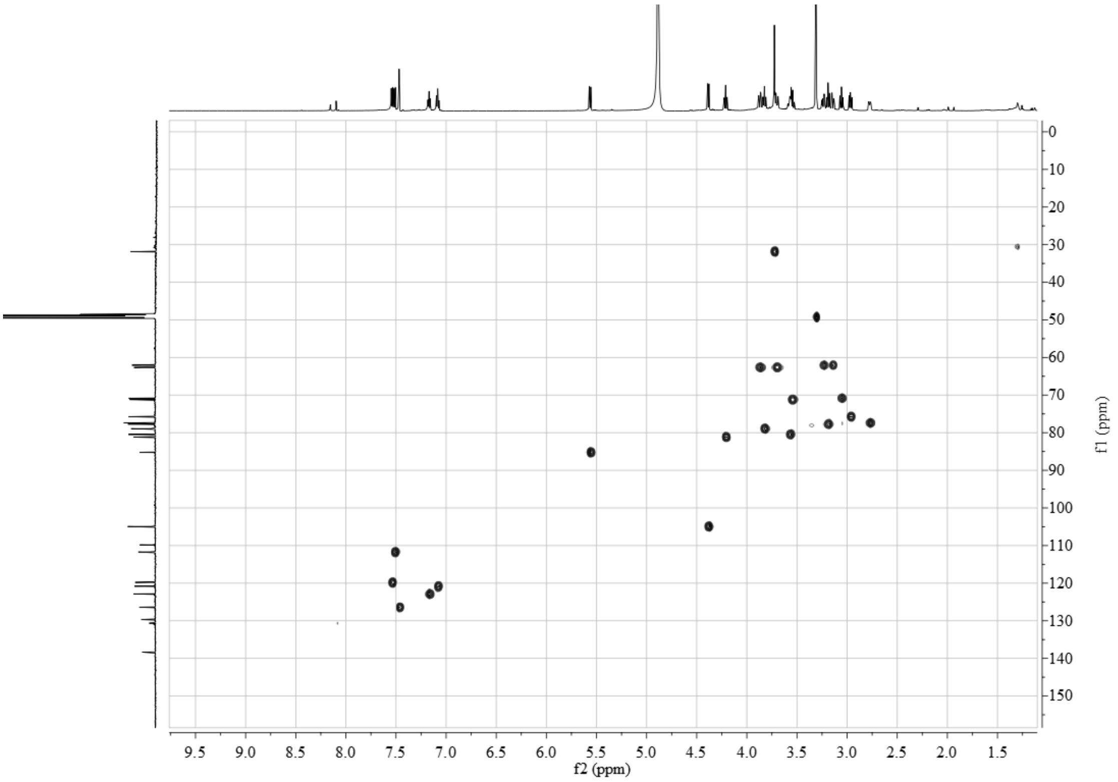

S39. HMBC Spectrum of the new compound **6**

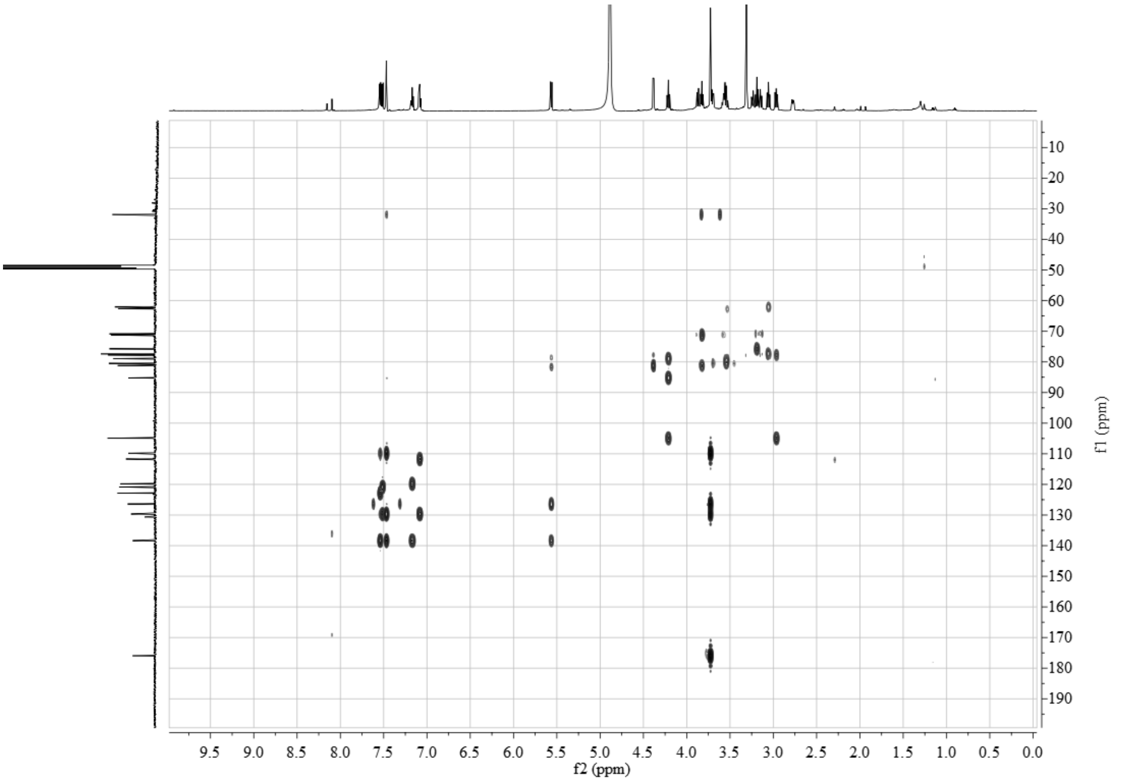

S40. HRESIMS spectrum of the new compound **6**

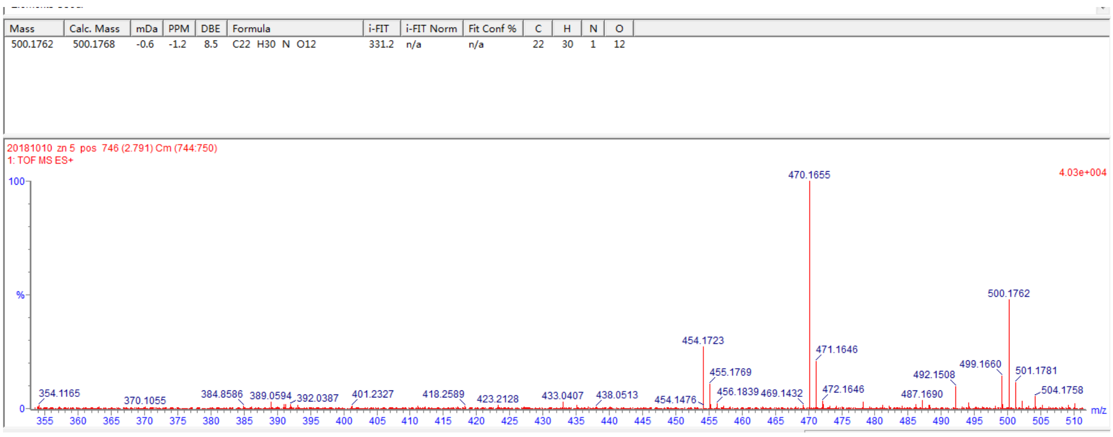

S41. UV spectrum of the new compound **6**

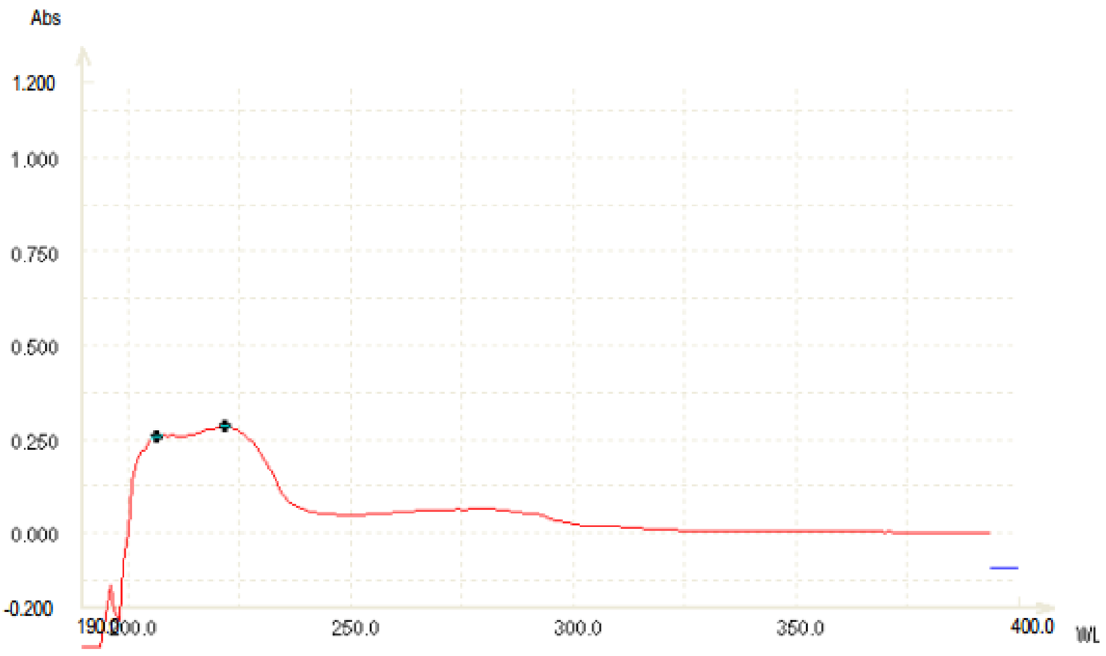

S42. IR spectrum of the new compound **6**

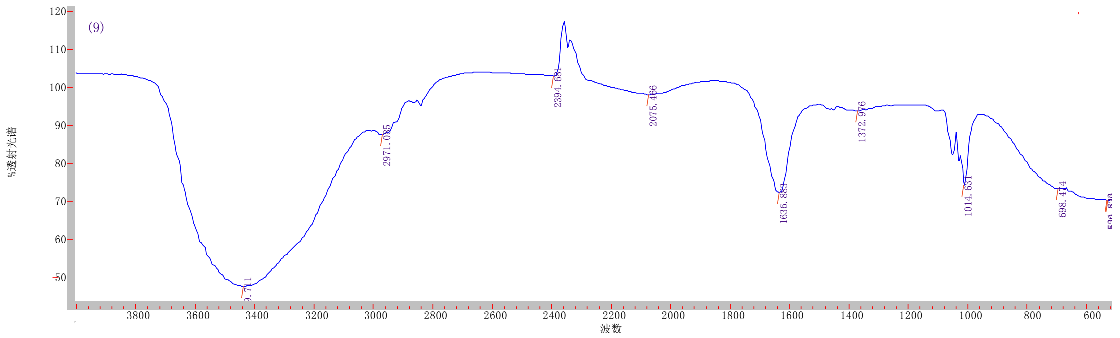

S43. Cytotoxic activities of compounds **1-10** on PC12 cells at 10 μM

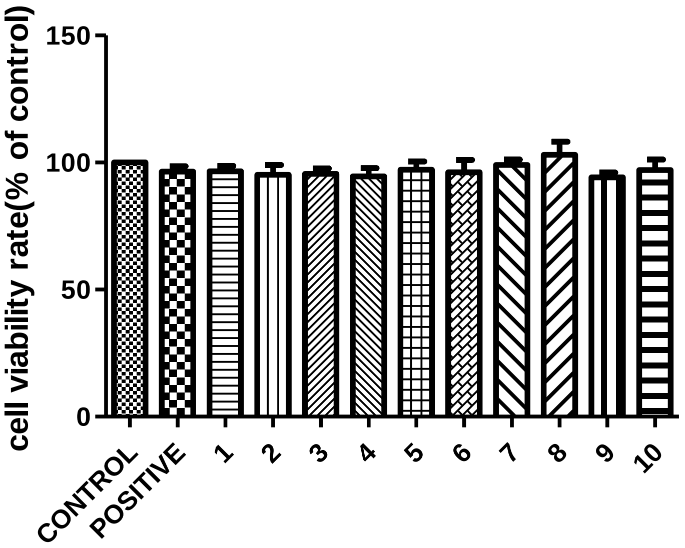

Table S1. Antitumor activities (IC_{50} μM, n = 3) of compounds **1-10** and 5-Fu. The data are expressed as means ± SEM.

	1	2	3	4	5	6	7	8	9	10	5-Fu
Hep G2	> 50	> 50	> 50	> 50	> 50	> 50	> 50	> 50	> 50	> 50	11.26 ± 0.83
HCT-116	> 50	> 50	> 50	> 50	> 50	> 50	> 50	> 50	> 50	> 50	4.54 ± 1.33
MGC-803	> 50	> 50	> 50	> 50	> 50	> 50	> 50	> 50	> 50	> 50	12.54 ± 1.07
